# Return on investment of community health workers in the United States: a systematic review

**DOI:** 10.1016/j.lana.2026.101469

**Published:** 2026-04-21

**Authors:** Muhammed Rashid, Max Fu, Pattranit Jitareewong, Jeong-Yeon Cho, Richard E. Nelson, Rachel M. Ceballos, Surasak Saokaew, Nathorn Chaiyakunapruk

**Affiliations:** a Department of Pharmacotherapy, College of Pharmacy, University of Utah, Salt Lake City, UT, USA; b Department of Social and Administrative Pharmacy, Faculty of Pharmaceutical Sciences, Chulalongkorn University, Bangkok, Thailand; c Department of Internal Medicine, University of Utah School of Medicine, Salt Lake City, UT, USA; d Department of Family Medicine and Public Health, University of Utah, Salt Lake City, UT, USA; e Division of Social and Administrative Pharmacy, Department of Pharmaceutical Care, School of Pharmaceutical Sciences, University of Phayao, Phayao, Thailand; f Unit of Excellence on Clinical Outcomes Research and Integration (UNICORN), School of Pharmaceutical Sciences, University of Phayao, Phayao, Thailand; g Faculty of Pharmacy, Silpakorn University, Nakhon Pathom, Thailand; h IDEAS Center, Veterans Affairs Salt Lake City Healthcare System, Salt Lake City, UT, USA

**Keywords:** Community health worker, Cost, Return on investment, Workforce studies

## Abstract

**Background:**

Community Health Workers (CHWs) play a vital role in improving health equity and patient outcomes in the United States (US). However, a systematic synthesis of their economic value remains limited. This review aimed to critically appraise the methodologies and consolidate the evidence on the return on investment (ROI) of CHW programmes in the US.

**Methods:**

We conducted a systematic review of US-based economic evaluations that assessed the ROI of CHW programmes. We searched PubMed, EMBASE, Web of Science, EconLit, CEA Registry, and available grey literature from inception to April 2025. Two reviewers independently screened studies, extracted data, and assessed quality using the NICE CBA Compilers checklist. Programme costs and savings were annualized and inflated to 2024 US dollars. Narrative synthesis was performed to summarise the evidence.

**Findings:**

Thirty-five studies (of 2155 records) representing 41 distinct CHW programmes across 23 states were included. The ROI analyses were commonly conducted in the Southern region, with 16 analyses (39%). Programmes most frequently targeted patients with diabetes and high admission risk (n = 8; 19.5%). Latino/Hispanic populations were the most frequently targeted (n = 8; 19.5%). The healthcare system perspective (n = 30; 73%) was predominantly adopted, with time horizons up to 20 years. Most ROI analyses (n = 37; 88%) used simple cost analysis, while 4 (9.7%) used Markov-modelling. The median inflation-adjusted annual programme cost was $155,275, yielding median annual savings of $403,298. The median ROI was $2.12 (interquartile range: 1.64–4.03) per dollar invested. Sensitivity and scenario analyses were conducted by 17 (48.5%) studies.

**Interpretation:**

CHW programmes in the US demonstrate consistent, favourable financial returns. Future research should consider standardized methodologies integrating equity metrics.

**Funding:**

The study was funded by a cooperative agreement CDC-RFA-FT-23-0069 from the CDC's Centre for Forecasting and Outbreak Analytics. The funders had no role in the conduct of this study, data collection, management, analysis, and interpretation of the data. Its contents are solely the responsibility of the authors and do not necessarily represent the official views of the Centers for Disease Control and Prevention.

**Research in context**

**Evidence before this study**

Community Health Workers (CHWs) have long been recognized as key contributors to improving health outcomes and reducing disparities in the United States (US). Despite growing investment in CHW programmes, evidence on their economic impact and return on investment (ROI) remains fragmented. Previous reviews have largely focused on health outcomes or the cost-effectiveness of CHW interventions but have not comprehensively appraised the methodological quality or synthesized the financial returns of these programmes. No prior review has consolidated evidence across diverse populations, disease areas, and geographic regions within the US using standardized economic appraisal criteria. We systematically reviewed the US-based economic evaluations that have assessed the ROI of CHW programmes. We searched PubMed, EMBASE, Web of Science, EconLit, the CEA Registry, and relevant grey literature databases from inception to April 2025 using combinations of terms related to “community health worker,” “economic evaluation,” “cost benefit,” and “return on investment.” The NICE CBA Compilers checklist was used for the quality assessment of evidence identified.

**Added value of this study**

This systematic review provides the most comprehensive synthesis to date of US-based CHW economic evaluations, encompassing 35 studies representing 41 distinct programmes across 23 states. By harmonizing costs and savings to 2024 US dollars and applying a structured critical appraisal framework, our review reveals that CHW programmes consistently generate positive financial returns, with a median ROI of $2.12 (IQR 1.64–4.03) per dollar invested. It also identifies key methodological variation across studies, such as differing perspectives, time horizons, and modelling approaches that influence ROI estimates. Importantly, our review highlights gaps in equity integration and sensitivity analysis, providing an evidence base to inform more rigorous, standardized future evaluations.

**Implications of all the available evidence**

CHW programmes offer substantial and consistent economic benefits alongside their well documented public health impact, supporting their broader adoption and sustained funding in the US healthcare system. However, variability in methodological approaches limits comparability across studies. Standardizing ROI evaluations and incorporating equity-focused metrics could enhance the credibility and policy relevance of future economic assessments. These findings reinforce the economic case for integrating CHWs as an important component of value-based care and health equity strategies nationwide.

## Introduction

Community Health Workers (CHWs) are frontline public health workers who serve as a crucial bridge between underserved populations and the healthcare system in the United States (US) and globally.^1^, ^2^, ^3^ CHWs are trained, skilled community members who work with communities to improve health through various strategies.^2^ They provide culturally competent education, advocacy, direct services such as chronic disease management, preventive care, health education, essential emotional or social support, and capacity building, significantly improving health outcomes and disparities.^2^^,^^4^ Their role is particularly vital in addressing the widespread healthcare access limitations across the nation; for instance, approximately 60 million Americans (18% of the population) reside in rural areas, where healthcare access is limited due to provider shortages, hospital closures, and geographic barriers.^5^, ^6^, ^7^ About 100 million people (30% of the US population) live in federally designated medically underserved areas and health professional shortage areas.^7^, ^8^, ^9^ CHWs effectively provide healthcare services in these communities, assisting governments in reaching these populations to improve healthcare access and health outcomes through building community trust.^1^^,^^10^^,^^11^

Despite growing interest and recognized healthcare impact, concerns about the return on investment (ROI) of CHW programmes remain a significant barrier to broader implementation and policy support.^1^^,^^12^, ^13^, ^14^ Traditional economic evaluations, such as cost-effectiveness and cost-utility analyses, compare costs and health outcomes across intervention alternatives.^15^ In contrast, ROI frameworks provide more practical and directly actionable metrics by translating programme value into monetary returns relative to investment.^16^^,^^17^ ROI analyses are increasingly employed in public health settings, with results highly relevant to public health officers guiding the budgetary decision-making, resource allocation, and policy advocacy.^16^, ^17^, ^18^ ROI is typically calculated as the ratio of net monetary benefits (e.g., healthcare cost savings, avoided utilization, or productivity gains) to programme implementation costs (e.g., personnel, supplies, travel etc.), expressed as dollars returned per dollar invested. The definition of “return” varies across studies and may include direct medical cost offsets, broader societal benefits, or both, while “investment” generally reflects programme delivery and operational costs.^12^^,^^17^ These ROI measures vary based on the perspective, intervention components, reimbursement systems, and analytical context.^19^, ^20^, ^21^

The most recent comprehensive analysis of CHW programmes in the US was conducted a decade ago. It did not incorporate ROI analyses, reporting only partial cost-effectiveness measures for some studies.^22^ Similarly, a recent scoping review^23^ of CHW programmes revealed significant methodological heterogeneity in cost metrics and outcome measures, making generalization and cross-setting comparisons difficult. This review focused on lower-middle-income countries (LMICs) and did not report ROI summary measures, limiting generalizability about the impact of CHWs in the US.

Current CHW programme funding gaps are estimated to be US $4.4 billion globally.^23^^,^^24^ In the US, a lack of uniform funding strategies and limited Medicaid reimbursement for CHW services present additional challenges.^25^ These funding gaps can severely disrupt CHW activities. In the context of growing interest in value-based public health strategies, understanding and synthesizing the evidence on CHW intervention ROI metrics of CHW interventions in the US is essential for informing actionable policy and funding decisions.

This review critically examines how ROI is defined, measured, and reported across studies, including cost components, outcome metrics, and analytical approaches for US CHW interventions. Through our findings from this study, we aim to strengthen the evidence base, guide future research, and support strategic resource allocation within the healthcare system and government settings by identifying best practices and methodological gaps.

## Methods

This systematic review is registered in PROSPERO (CRD42023456789) and reported following the Preferred Reporting Items for Systematic reviews and Meta-Analysis (PRISMA) 2020 checklist.^26^

### Literature search

We conducted comprehensive literature searches in PubMed, EMBASE, and Web of Science from inception to April 2025, focusing on the key concepts: “ROI” & “Community Health Workers” & “United States”. We did not apply any date or language restrictions in the literature search. Additional sources such as the EconLit database, the Cost-Effectiveness Analysis (CEA) Registry, websites of state and local health departments, and national associations including National Association of County and City Health Officials (NACCHO), National Association of Community Health Workers (NACHW), and Health Resources & Services Administration (HRSA). Bibliographic searches of selected articles identified additional relevant literature. Detailed search strategy is provided in Supplementary File S1.

### Eligibility criteria

Studies were included if they met following criteria: (i) considered any US populations; (ii) assessed the ROI of CHW interventions including cost analysis or economic impact modelled after randomized controlled trials (RCTs), observational studies or literature evidence; (iii) used any comparators to assess the clinical and cost benefits; (iv) reported ROI or provided sufficient data for ROI calculation (e.g., cost savings from CHW intervention, and total cost of the intervention programme); and (v) were an original economic evaluation calculating ROI. No timeframe restrictions were applied to the eligibility criteria. Studies from outside the US and those not reporting CHW interventions were excluded.

### Study selection and data extraction

Title/abstract screening followed by full-text screening were conducted in Covidence^27^ according to predefined criteria. Data were extracted into a predefined data extraction grid in Microsoft Excel. Extracted data included publication characteristics, intervention details (CHW programmes, roles, target populations, community settings, programme duration, outcome assessment), intervention cost components, ROI methodology (perspective, case load assumptions, economic modelling used, discounting, time horizon, total cost, ROI values, sensitivity and scenario analysis). Two independent reviewers (MF, PJ) performed study selection and data extraction, with discrepancies were resolved by consensus or a third reviewer (MR, SS).

### Quality assessment

Two independent reviewers (MF, PJ) assessed the quality of studies using the CBA Compilers of NICE Public Health Reviews checklist.^28^ The NICE CBA Compilers checklist provides structured, transparent, and reliable assessment of ROI studies compared to general economic evaluation checklists.^29^, ^30^, ^31^ This 17-items tools covers domains including research question, comparator, perspective, credibility and accuracy of cost and outcome assessment, cost adjustment, net values, assumptions, sensitivity analyses, result generalizability, and equity. A total score from 1 to 17 was assigned by giving 1 for “yes” and 0 for “no” answer. We descriptively summarized all the methodological issues or missing data that resulted in a score of “0” for any checklist domain. Disagreements were resolved by consensus or third reviewer (MR).

### Evidence synthesis

Narrative synthesis was conducted with key findings summarized in tables. CHW roles were grouped into seven categories according to standard core roles and competencies outlined by Rosenthal et al.^2^ This includes (i) bridging or providing cultural mediation between communities and the health care systems, (ii) offering health education and information that is culturally appropriate and accessible, (iii). making sure that people receive the services they need; (iv) giving informal counselling and social support, (v) advocating for the needs of individuals and communities, (vi) delivering direct services, and (vii) building the capacity of individuals and communities. Details of activities under each category are provided in Supplementary File S2. Geographic regions were categorized based on the CDC recommendations for US health.^32^ All cost estimates were annualized and inflated to 2024 USD using the latest available annual average Consumer Price Index (CPI) from US Bureau of Labor Statistics^33^ to facilitate the analysis and ensure uniformity. Cost data are presented as medians with interquartile range (IQR).

All the analyses were performed in Microsoft Excel. As ROI values were not normally distributed, the results are presented as medians with interquartile ranges. We performed a nonparametric bivariate comparison (Mann–Whitney U test for two groups and Kruskal–Wallis test for three or more groups) to explore whether program characteristics (CHW roles, regions, target population, and program duration), and methodological feature (perspective and conduct of sensitivity analysis) were associated with higher ROI. A meta-analysis was not performed due to substantial heterogeneity in intervention design, target populations, and ROI methodologies.

### Role of the funding source

This study was not funded, and the funders had no role in study design, data collection, data analysis, interpretation, writing of the report.

## Results

### Study selection process

**Fig. 1:**
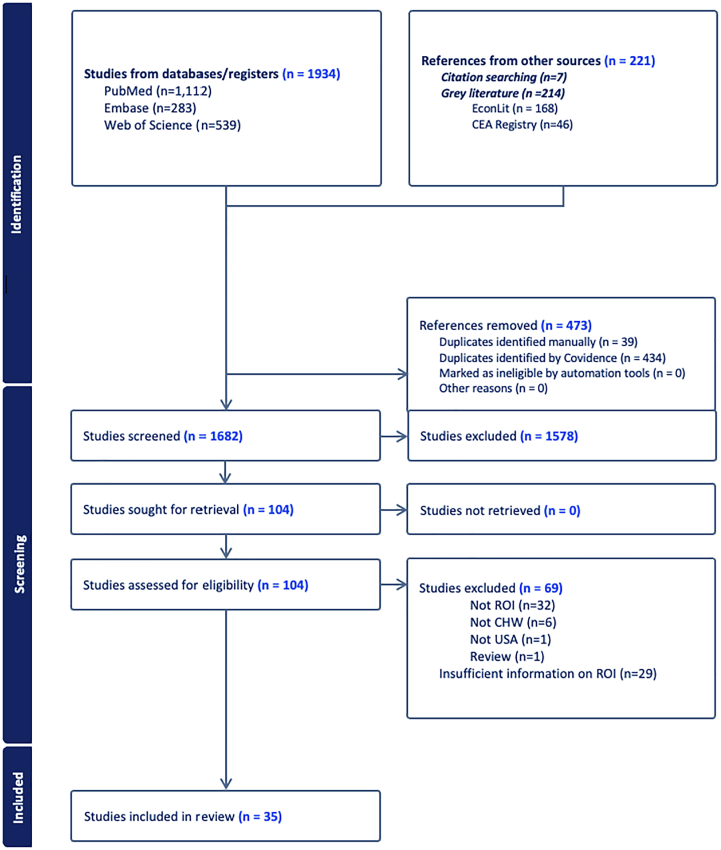
**The PRISMA flow diagram for study selection**. This diagram illustrates the process of study identification, screening, eligibility assessment, and inclusion in the review in accordance with PRISMA guidelines.

Literature searches identified 2155 records, with 1682 studies screened after removing 473 duplicates. Of these, 104 studies were assessed for eligibility, yielding 35 included studies.^12^^,^^19^, ^20^, ^21^^,^^34^, ^35^, ^36^, ^37^, ^38^, ^39^, ^40^, ^41^, ^42^, ^43^, ^44^, ^45^, ^46^, ^47^, ^48^, ^49^, ^50^, ^51^, ^52^, ^53^, ^54^, ^55^, ^56^, ^57^, ^58^, ^59^, ^60^, ^61^, ^62^, ^63^, ^64^ Sixty-nine studies were excluded for the following reasons: 32 did not report ROI, 29 provided insufficient information to calculate ROI, 6 did not involve CHW intervention, 1 was conducted outside the US, and 1 was a review article. Detailed PRISMA flow diagram is provided in Fig. 1, with excluded studies listed in Supplementary File S3.

### Characteristics of included studies

Thirty-five studies^12^^,^^19^, ^20^, ^21^^,^^34^, ^35^, ^36^, ^37^, ^38^, ^39^, ^40^, ^41^, ^42^, ^43^, ^44^, ^45^, ^46^, ^47^, ^48^, ^49^, ^50^, ^51^, ^52^, ^53^, ^54^, ^55^, ^56^, ^57^, ^58^, ^59^, ^60^, ^61^, ^62^, ^63^, ^64^ published between 2006 and 2025 were included, evaluating 41 different CHW programmes. Two studies by London et al.,^34^ and Gillam et al.,^21^ analysed four different CHW programmes in individual reports. Four studies^21^^,^^34^^,^^62^^,^^63^ were organizational reports (Table 1).

**Table 1:** Summary characteristics of included analyses (n = 41).

Characteristic	No. of analyses (n, %)	References
US Geographical region		
South	16 (39.02%)	^20^^,^^21^^,^^35^^,^^36^^,^^39^^,^^42^^,^^47^^,^^48^^,^^51^^,^^52^^,^^55^^,^^57^^,^^59^
Northeast	12 (29.27%)	^12^^,^^19^^,^^34^^,^^43^^,^^44^^,^^46^^,^^53^^,^^54^^,^^58^
West	7 (17.07%)	^37^^,^^38^^,^^40^^,^^49^^,^^56^^,^^62^^,^^64^
Midwest	6 (14.63%)	^41^^,^^45^^,^^50^^,^^60^^,^^61^^,^^63^
Health condition		
Diabetes	8 (19.5%)	^21^^,^^34^^,^^36^^,^^37^^,^^47^^,^^49^^,^^55^^,^^61^
Risk of readmission/ED visit	8 (19.5%)	^20^^,^^21^^,^^43^^,^^48^^,^^51^^,^^57^^,^^58^^,^^62^
Paediatric asthma	7 (17.1%)	^19^^,^^34^^,^^42^^,^^44^^,^^46^^,^^50^^,^^64^
Cancer	5 (12.2%)	^39^^,^^40^^,^^52^^,^^56^^,^^59^
Multiple chronic diseases	3 (7.3%)	^12^^,^^21^^,^^63^
Pregnancy	2 (4.9%)	^21^^,^^41^
Cardiovascular diseases	2 (4.9%)	^34^^,^^45^
Dementia	1 (2.4%)	^35^
HIV	1 (2.4%)	^60^
Other patient population	4 (9.7%)	^21^^,^^38^^,^^53^^,^^54^
Community settings		
Latino/Hispanic	8 (19.5%)	^19^^,^^34^^,^^36^^,^^39^^,^^42^^,^^47^^,^^52^
Rural	3 (7.5%)	^34^^,^^41^^,^^60^
Black/African American	3 (7.3%)	^19^^,^^34^^,^^50^
Underserved	2 (5%)	^38^^,^^56^
Low-Socioeconomic Urban	1 (2.5%)	^45^
American Samoa	1 (2.5%)	^49^
Tech-Naïve	1 (2.5%)	^54^
Unemployed	1 (2.5%)	^59^
Role titles for CHWs		
CHWs	31 (75.61%)	^12^^,^^19^^,^^21^^,^^34^, ^35^, ^36^, ^37^, ^38^^,^^41^, ^42^, ^43^, ^44^, ^45^^,^^47^, ^48^, ^49^, ^50^, ^51^, ^52^, ^53^^,^^55^^,^^56^^,^^61^, ^62^, ^63^, ^64^
Patient navigators	6 (14.63%)	^39^^,^^52^^,^^54^^,^^58^, ^59^, ^60^
Lay health workers	2 (5%)	^20^^,^^40^
Home health worker	1 (2.5%)	^46^
Grand aides	1 (2.5%)	^57^
Community or stakeholder engagement		
Design and planning of intervention		
Patient interview or validated questionnaire assessment to understand the need	3 (7.3%)	^12^^,^^20^^,^^43^
Healthcare stakeholder interview to plan CHW programs	1 (2.4%)	^43^
Implementation and execution of intervention		
Trusted culturally matched CHW	7 (17.7%)	^34^^,^^36^^,^^49^^,^^50^^,^^55^^,^^58^^,^^61^
Academic & rural community advisory board collaboration	3 (7.3%)	^41^^,^^46^^,^^53^
Community & interdepartmental academic collaboration	2 (5%)	^43^^,^^60^
Academic & local CHW organizations or networks collaboration	4 (9.7%)	^46^^,^^47^^,^^53^^,^^60^
Perspective of ROI analysis		
Healthcare system	30 (73.17%)	^20^^,^^21^^,^^34^^,^^36^^,^^38^, ^39^, ^40^, ^41^^,^^43^^,^^45^^,^^46^^,^^48^^,^^51^^,^^53^, ^54^, ^55^, ^56^, ^57^, ^58^, ^59^, ^60^, ^61^, ^62^, ^63^
Societal perspective	8 (19.5%)	^34^^,^^36^^,^^42^^,^^46^^,^^47^^,^^49^^,^^52^^,^^55^^,^^60^
Payer perspective	16 (39%)	^12^^,^^19^^,^^21^^,^^34^^,^^35^^,^^37^^,^^38^^,^^44^^,^^50^^,^^64^
Components of cost of intervention		
Personnel cost	40 (97.6%)	^12^^,^^19^, ^20^, ^21^^,^^34^, ^35^, ^36^, ^37^, ^38^, ^39^, ^40^, ^41^, ^42^, ^43^, ^44^, ^45^^,^^47^, ^48^, ^49^, ^50^, ^51^, ^52^, ^53^, ^54^, ^55^, ^56^, ^57^, ^58^, ^59^, ^60^, ^61^, ^62^, ^63^, ^64^
CHW training cost	32 (78%)	^12^^,^^19^^,^^21^^,^^34^, ^35^, ^36^^,^^39^, ^40^, ^41^, ^42^, ^43^^,^^45^^,^^47^^,^^48^^,^^50^^,^^51^^,^^53^, ^54^, ^55^^,^^57^^,^^59^, ^60^, ^61^, ^62^, ^63^, ^64^
Non-personnel cost	30 (73.2%)	^12^^,^^19^^,^^21^^,^^34^, ^35^, ^36^, ^37^, ^38^, ^39^, ^40^, ^41^, ^42^^,^^44^^,^^46^^,^^47^^,^^49^^,^^52^^,^^55^, ^56^, ^57^, ^58^^,^^60^^,^^62^^,^^64^
Indirect cost	7 (17%)	^12^^,^^37^^,^^45^^,^^47^^,^^49^^,^^55^^,^^60^
Primary data used for the outcome assessment		
Pre-post intervention Studies	19 (46.3%)	^19^^,^^20^^,^^34^^,^^35^^,^^37^^,^^38^^,^^40^^,^^42^^,^^46^^,^^48^^,^^53^^,^^60^^,^^61^
Non-randomized intervention studies	10 (24.4%)	^36^^,^^39^^,^^41^^,^^45^^,^^50^^,^^51^^,^^54^^,^^59^^,^^62^^,^^63^
RCTs	7 (17.1%)	^12^^,^^43^^,^^49^^,^^55^^,^^56^^,^^58^^,^^64^
Observational studies	4 (9.8%)	^44^^,^^47^^,^^52^^,^^57^
Literature evidence considered for analysis	25 (61.0%)	^12^^,^^19^^,^^21^^,^^34^, ^35^, ^36^, ^37^, ^38^, ^39^^,^^47^^,^^49^^,^^52^^,^^56^^,^^58^^,^^60^^,^^62^, ^63^, ^64^
Outcomes considered in the analysis		
Healthcare utilization	37 (90.2%)	^12^^,^^19^, ^20^, ^21^^,^^34^^,^^35^^,^^37^, ^38^, ^39^, ^40^, ^41^, ^42^, ^43^, ^44^, ^45^, ^46^, ^47^, ^48^, ^49^, ^50^, ^51^, ^52^^,^^54^, ^55^, ^56^, ^57^, ^58^, ^59^^,^^61^, ^62^, ^63^, ^64^
Clinical outcomes	30 (73.2%)	^12^^,^^19^, ^20^, ^21^^,^^34^^,^^36^^,^^37^^,^^39^, ^40^, ^41^, ^42^, ^43^, ^44^, ^45^^,^^47^, ^48^, ^49^, ^50^^,^^52^^,^^55^^,^^60^^,^^61^^,^^64^
Loss of productivity	8 (19.5%)	^34^^,^^42^^,^^47^^,^^49^^,^^55^
Improved hospital and insurance access	7 (17.1%)	^37^^,^^39^^,^^41^^,^^43^^,^^54^^,^^56^^,^^57^
Model and analysis used		
Simple cost analysis	37 (87.9%)	^12^^,^^19^, ^20^, ^21^^,^^34^^,^^35^^,^^37^^,^^38^^,^^40^, ^41^, ^42^, ^43^, ^44^^,^^46^, ^47^, ^48^, ^49^, ^50^, ^51^^,^^53^, ^54^, ^55^, ^56^, ^57^, ^58^, ^59^, ^60^, ^61^, ^62^, ^63^, ^64^
Markov modelling	4 (9.7%)	^36^^,^^39^^,^^45^^,^^52^
Monte-Carlo simulation	1 (2.4%)	^36^
Discount rate reported	9 (24.4%)	^34^^,^^36^^,^^37^^,^^39^^,^^47^^,^^52^
Sensitivity analyses		
1-way sensitivity analysis	11 (26.8%)	^20^^,^^35^^,^^36^^,^^39^^,^^41^^,^^42^^,^^47^^,^^49^^,^^54^^,^^63^^,^^64^
2-way sensitivity analysis	3 (7.3%)	^12^^,^^39^^,^^52^
Probabilistic sensitivity analysis (PSA)	2 (4.9%)	^36^^,^^56^
Scenario analysis	5 (12.2%)	^19^^,^^41^^,^^43^^,^^57^^,^^64^

CHW: Community health worker; ED: Emergency department; HIV: Human Immunodeficiency Virus; RCT: Randomized controlled trial; US: United States.

### Geographical distribution of studies

**Fig. 2:**
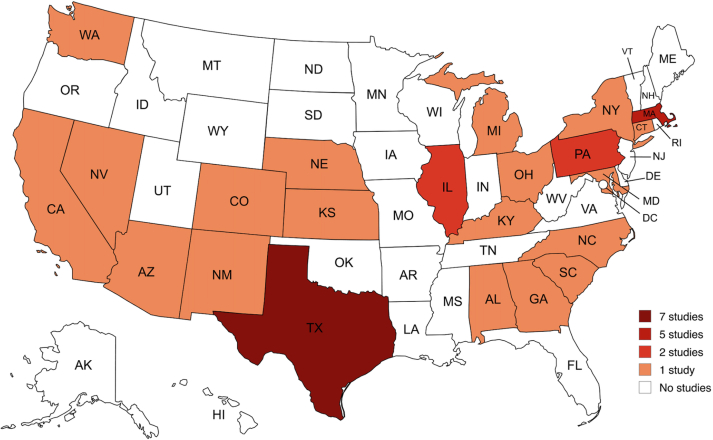
**Geographical distribution of included studies across the United States**. This map illustrates the number of included studies conducted in each U.S. state. States are shaded according to the number of studies identified: dark red indicates 7 studies, red indicates 5 studies, orange indicates 2 studies, light orange indicates 1 study, and white indicates no studies. The map includes all U.S. states to provide a complete geographical overview.

Twenty-three states were represented in CHW intervention ROI analyses, with the largest proportion of studies coming from the Southwestern and Southeastern regions of the US (n = 16; 39.02%). Texas (n = 7; 17.1%) and Massachusetts (n = 5; 12.2%) were the most frequently represented states. Parts of the Midwest were represented, while large geographical areas, including the Mountain West, the Pacific Northwest, and several Deep Southern states, had few or no studies examining the financial return of CHW interventions (Fig. 2).

### Quality assessment of included studies

Mean score for quality assessment was 12.66 out of 17 (range 8^38^ to 15^21^^,^^39^). Several methodological concerns were identified. Fifteen studies (42.8%)^36^, ^37^, ^38^, ^39^, ^40^^,^^42^^,^^46^^,^^48^^,^^51^^,^^53^^,^^54^^,^^58^, ^59^, ^60^, ^61^ failed to explicitly state the analysis perspective, requiring reviewers’ interpretation. Twenty-three studies (65.7%)^12^^,^^19^^,^^20^^,^^34^, ^35^, ^36^^,^^39^^,^^40^^,^^42^, ^43^, ^44^^,^^46^^,^^47^^,^^49^^,^^50^^,^^52^^,^^53^^,^^55^, ^56^, ^57^^,^^60^^,^^61^^,^^64^ did not quantify costs and outcomes in monetary terms for comparator arms, making incremental cost-return assessment difficult. Adjustments for differential timing (n = 29; 82.8%)^12^^,^^19^^,^^20^^,^^34^^,^^35^^,^^38^^,^^40^, ^41^, ^42^, ^43^, ^44^, ^45^, ^46^^,^^48^, ^49^, ^50^, ^51^^,^^53^, ^54^, ^55^, ^56^, ^57^, ^58^, ^59^, ^60^, ^61^, ^62^, ^63^, ^64^ and net-present value reporting (n = 16; 45.7%)^38^^,^^40^^,^^45^, ^46^, ^47^, ^48^, ^49^^,^^51^, ^52^, ^53^^,^^55^, ^56^, ^57^, ^58^, ^59^, ^60^ were often missing, reducing temporal accuracy and comparability. While 17 studies (48.5%)^12^^,^^19^^,^^20^^,^^35^^,^^36^^,^^39^^,^^41^, ^42^, ^43^^,^^47^^,^^49^^,^^52^^,^^54^^,^^56^^,^^57^^,^^63^^,^^64^ conducted sensitivity or scenario analysis, the remaining 18 did not, limiting understanding of the robustness of their findings under varying conditions. Only Wilson et al., published in 2015, discussed the distributional or equity aspect of CHW intervention through targeting the interventions to Hispanic men (which address the ethnic and gender dimension), however a formal analysis was not performed.^39^ Detailed quality assessment is provided in Supplementary File S4.

### Characteristics of CHW interventions and roles

**Fig. 3:**
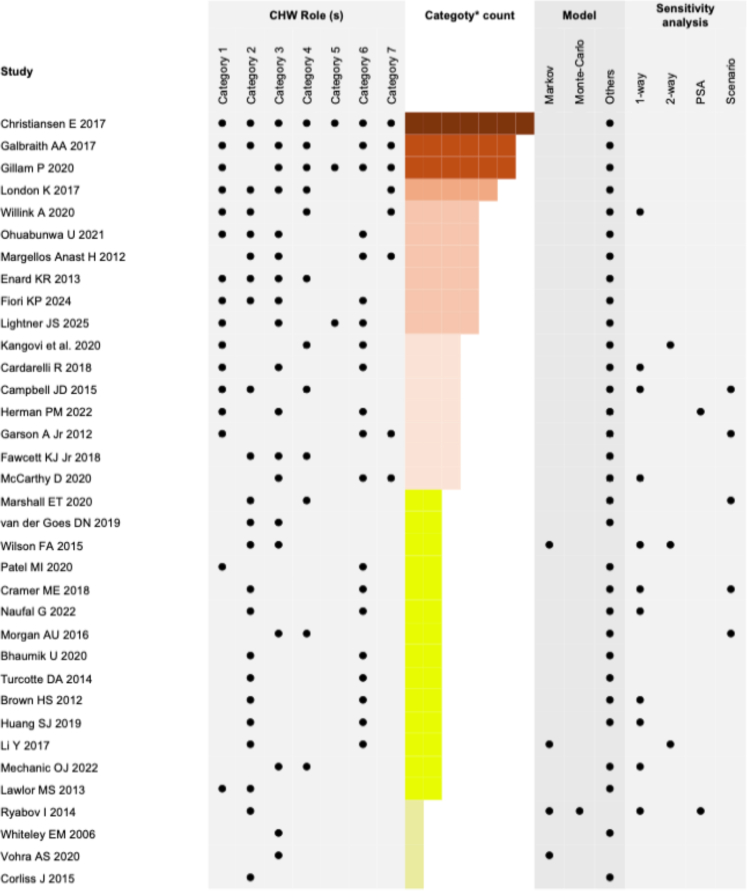
**Distribution of Community Health Worker (CHW) roles, model types, and sensitivity analyses across included studies**. This figure presents the distribution of CHW roles, economic evaluation models, and types of sensitivity analyses used across the included studies. Each row represents an individual study. Black dots indicate the presence of a specific CHW role category, model type and sensitivity analysis method in each study. The colour gradient reflects the number of CHW role categories included in each study, with darker shades indicating a higher number of categories. CHW roles are grouped into seven predefined categories based on standard classifications.

Across the included studies, CHWs fulfilled multiple roles that fall into the seven categories described by Rosenthal^2^ and based on core competencies and services they provide. Fourteen studies (40%)^19^^,^^37^^,^^39^, ^40^, ^41^, ^42^, ^43^, ^44^^,^^46^^,^^47^^,^^49^^,^^52^^,^^54^^,^^55^ included two CHW role categories. Only Christiansen et al.^62^ covered all seven CHW roles, while Galbraith et al.^58^ and Gillam et al.^21^ addressed six categories. London et al.^34^ reported CHW roles under five domains, and six studies (17.1%)^35^^,^^48^^,^^50^^,^^51^^,^^53^^,^^60^ covered four CHW role domains, while 7 studies (20%)^12^^,^^20^^,^^35^^,^^56^^,^^57^^,^^61^^,^^63^^,^^64^ focused on three categories. Four studies (11.4%)^36^^,^^38^^,^^45^^,^^59^ considered only single primary CHW role (Fig. 3). Sixteen studies (45.7%)^12^^,^^20^^,^^34^^,^^35^^,^^40^^,^^48^^,^^51^^,^^53^^,^^55^, ^56^, ^57^, ^58^^,^^60^^,^^62^^,^^64^ described CHWs acting as cultural mediators between communities and the health care systems through community education on healthcare service use, needs assessment, information gathering, interpretation, and translation. Providing culturally appropriate health education on health promotion, prevention, and chronic illness management was evident in 23 studies (65.7%).^19^^,^^34^, ^35^, ^36^, ^37^^,^^39^^,^^41^^,^^42^^,^^44^^,^^46^, ^47^, ^48^, ^49^, ^50^, ^51^, ^52^, ^53^^,^^55^^,^^58^^,^^59^^,^^61^^,^^62^ Nineteen studies (54.3%)^20^^,^^21^^,^^34^^,^^37^, ^38^, ^39^^,^^43^^,^^45^^,^^48^^,^^50^^,^^51^^,^^53^^,^^54^^,^^56^^,^^58^^,^^60^, ^61^, ^62^ documented CHWs ensuring service access through patient identification, referrals, motivation, encouragement for care seeking, transportation to healthcare facilities, and follow-up. Twelve studies (34.3%)^12^^,^^19^^,^^21^^,^^34^^,^^35^^,^^43^^,^^51^^,^^54^^,^^58^^,^^61^^,^^62^^,^^64^ demonstrated CHWs involvement in information counselling and social support. Provision of direct clinical services, disease screening, and daily needs, including housing, were reported in 20 studies (57.1%).^12^^,^^20^^,^^21^^,^^40^, ^41^, ^42^^,^^44^^,^^46^, ^47^, ^48^, ^49^, ^50^^,^^52^^,^^53^^,^^56^, ^57^, ^58^^,^^60^^,^^62^^,^^63^ Individual and community capacity building was focused on eight studies (22.8%),^21^^,^^34^^,^^35^^,^^50^^,^^57^^,^^58^^,^^62^^,^^63^ while only 3 studies (8.6%)^21^^,^^60^^,^^62^ recorded the CHW patient advocacy role. Intervention duration ranged from 2 weeks^43^ to 3 years.^52^ Twenty-six studies (74.3%)^12^^,^^19^^,^^21^^,^^34^, ^35^, ^36^^,^^39^, ^40^, ^41^, ^42^, ^43^^,^^45^^,^^47^^,^^48^^,^^50^^,^^51^^,^^53^, ^54^, ^55^^,^^57^^,^^59^, ^60^, ^61^, ^62^, ^63^, ^64^ mentioned CHW training (Table 1). Detailed CHW roles categories are depicted in Fig. 3.

### CHW targeted population and setting

The included studies used the term CHW interchangeably with lay health workers,^20^^,^^40^ patient navigators,^39^^,^^52^^,^^54^^,^^58^, ^59^, ^60^ home health workers,^46^ and grand-aides,^57^ depending on the specific contexts. Interventions primarily targeted diabetic patients (n = 8; 19.5%),^21^^,^^34^^,^^36^^,^^37^^,^^47^^,^^49^^,^^55^^,^^61^ patients with high readmission or ED visits risk (n = 8; 19.5%),^20^^,^^21^^,^^43^^,^^48^^,^^51^^,^^57^^,^^58^^,^^62^ and children with asthma (n = 7; 17.1%).^19^^,^^34^^,^^42^^,^^44^^,^^46^^,^^50^^,^^64^ Eighteen programmes (43.9%) targeted underserved communities including Latino/Hispanic (n = 8; 19.5),^19^^,^^34^^,^^36^^,^^39^^,^^42^^,^^47^^,^^52^ rural (n = 3; 7.5%),^34^^,^^41^^,^^60^ Black/African American (n = 3; 7.5%),^19^^,^^34^^,^^50^ underserved neighbourhood (n = 2; 5%),^38^^,^^56^ low-Socioeconomic urban population (n = 1; 2.5%),^45^ and American Samoa (n = 1; 2.5%).^49^ One study each focused on tech-naïve^54^ and unemployed^59^ population (Tables 1 and 2).

**Table 2:** Characteristics of included studies.

Author, year	State	Target population	Community setting	CHW program and services	Comparator	Training for CHW	Primary data used & sample size	Duration of CHW program	Quality assessment score (out of 17)
Kangovi S 2020^12^	Pennsylvania	Patients with at least two chronic diseases	High-poverty neighborhood	IMPACT Program (Tailored social support such as housing instability, food insecurity, and limited social support)	Collaborative goal setting without CHW	Yes	RCT (I: 150; C: 152)^65^	6 months	14
London K 2017^34^	Connecticut	Children with uncontrolled Asthma	Latino (primarily Puerto Rican)	UTCO Program (Home visits, environmental assessment, mitigation supplies and education)	Usual care for Asthma and resources for self-management	Yes	Pre-post intervention study (I: 96; C: 96)^66^^,^^67^	4 months	14
		Patients with Type 2-Diabetes	Latinos (mostly Puerto Rican) and Black	Seattle–King County Medicaid Healthy Homes (Home visits, counseling, group education, and exercise classes)	Usual medical care at the clinics	Yes	Pre-post intervention study (I: 158; C: 158)^68^	18 months	14
		Individuals with Complex health needs	–	Molina Healthcare, CARE NM, Program (Healthcare access, schedule appointments, pain management and social support)	No CHW program	Yes	Pre-post intervention study (I: 72; C: 72)^69^	6 months	14
		Patients with CVD complications	Rural, underserved population	COACH Program (Diet modification, stress management, smoking cessation, exercise and medication management)	Usual care	Yes	Pre-post intervention study (I: 148; C: 184 patients)^70^	12 months	14
Willink A 2020^35^	Maryland	People with dementia	–	MIND Program (Home visits-based dementia care and coordination)	Usual care	Yes	Pre-post intervention study (I: 342; C: 120 patients)^71^	16 months	14
Marshall ET 2020^19^	Massachusetts	Children with uncontrolled Asthma	Low-income Hispanic and non-Hispanic Black families	READY Program (Home visits, Phone calls, self-management education, environmental trigger remediation education, and low-cost trigger remediation supplies)	No CHW program	Yes	Pre-post intervention study (ITT: 254)^72^	6 months	13
Ryabov I 2014^36^	Texas	Diabetic patients aged 30 or above	Mexican Americans	Monthly home visit and education	No CHW program	Yes	Experimental design (I: 15; C: 15)^47^^,^^73^	24 months	14
van der Goes DN 2019^37^	New Mexico	Patients with behavioral health disease, diabetes, obesity and tobacco use	–	Patient access (are coordinators and providing direct outreach to patients) in addition to other CHW activities (phone call, navigation, appointment scheduling education)	No CHW program	Yes	Pre-post intervention study (96,291 patients)^74^	12 months	15
Whitley EM 2006^38^	Colorado	Residents in Denver neighborhoods and special populations	Underserved community	MHI Program (Social support)	No CHW program	No	Intervention study (I: 590; C: 651 patients)^75^	18 months	8
Wilson FA 2015^39^	Texas	Men aged 50 years or older who had not received CRC screening in the last 10 years	Hispanic men who were members of Care Link (Bexar County's financial assistance program)	CCMN Program (Address language barriers, transportation and scheduling assistance, colon cancer, and screening knowledges)	Usual care without a navigator	Yes	Intervention (461 patients)	24 months	15
Patel MI 2020^40^	California	Patients with 1-year prior diagnosis of solid or hematologic malignant neoplasms	–	LHW-led Symptom Screening and Referral Intervention (Symptom screening and proactive referral intervention)	Usual care without an LHW	Yes	Pre-post multisite quality improvement study (I: 425; C: 407 patients)	12 months	9
Cramer ME 2018^41^	Nebraska	Pregnant women who speak and read either Spanish or English	Rural population	PTP with CHW Reinforcement (Weekly contact, prenatal care, mobile phone assisted education)	Usual prenatal care and printed educational materials	Yes	Two group experimental design (I: 41; C:36)	5.5 months	15
Cardarelli R 2018^20^	Kentucky	Medical/surgical admitted patients with high risk of being readmitted within 30 days based on a LACE score of 7 or greater	–	BTH Program (Patient centered care plan, addressing social needs, healthcare access and a follow-up call after the discharge)	No LHW program	Yes	Pre/Post Intervention quasi experimental study (I: 61; Baseline: 46 patients)^76^	5 months	13
Naufal G 2022^42^	Texas	Children with asthma diagnosed by their physician	Hispanic Children	AHHP Program (Home visits and asthma education)	No CHW program	Yes	Pre/Post Intervention (349 patients)	6 months	13
Morgan AU 2016^43^	Pennsylvania	Patients with preventable readmission	Low-income patients with limited access	IMPACT Program (Tailored social support such as housing instability, food insecurity, and limited social support)	No CHW program	Yes	RCT (446 patients)^77^, ^78^	2 weeks	12
Bhaumik U 2020^44^	Massachusetts	Children with higher asthma severity and/or social and environmental need with previous hospitalization in last 1-year	Low-income neighborhood	Community Asthma Initiative (Home visits to improve the understanding and adherence to asthma control measures, reduce the exposure and provide the support)	Usual care	NR	Observational data (I: 45; C:45patients)^79^	12 months	13
Vohra AS 2020^45^	Illinois	Patients with heart failure	Low- socioeconomicurban minority	Homes visits to assess vital signs and questions regarding well-being, weight management, symptoms, and medication adherence and phone calls	No CHW program	Yes	Intervention study cohort (I: 28; C: 28 patients)^80^	12 months	13
Turcotte DA 2014^46^	Massachusetts	Children under 15 years with asthma	Low- socioeconomic, urban households	Lowell Healthy Homes Program (Home visits for health and environmental assessment and customized remediation plan, pest management, commercial cleaning, providing healthy home cleaning equipment and supplies)	No CHW program	NR	Pre-post observational study (170 patients)	12 months	10
Brown HS 3rd, 2012^47^	Texas	Adults with type 2 diabetes	Low-income Hispanic adults	UTCO Program (Home visits, classroom health education classes, nutrition classes, exercise classes, and counseling sessions)	No CHW program	Yes	Simulated US representative sample (6551 patients)	18 months	14
Ohuabunwa U 2021^48^	Georgia	Patients who had hospitalization or visited the ER up to three times within a 90-day period, and identified to be at high risk for readmission	–	CHOOSE Health™ Program (Home visits, phone outreach, pharmaceutical care plan, in-hospital visits accompaniment, social services and self-management education)	No CHW program	Yes	Pre-post intervention (154 patients)	12 months	12
Campbell JD 2015^64^	Washington	Children with provider-diagnosed not well controlled or very poorly controlled asthma and enrolled in 1 of 2 Medicaid plans	–	Seattle–King County Medicaid Healthy Homes (Home visits, telephone outreach and e-mail-based support, education, support, and service coordination)	Usual care	Yes	RCT (I: 182; C: 191 patients)	12 months	14
Huang SJ 2019^49^	American Samoa	Type-2 diabetes patients	American Samoans	DCAS Program (Home visit, help to keep health care appointments, patients understand diabetes, reinforced adherence to medication regimens, problem-solved barriers to self-care, provided support, and mobilized family support for diabetes self-management)	Standard care	Yes	RCT (I: 104 C: 164 patients)^81^	12 months	13
Margellos-Anast H 2012^50^	Illinois	Children aged 2–16 years with severe, poorly controlled asthma per the National Heart, Lung, and Blood Institute standards	African American children	Home visits with tailored asthma education, trigger assessment, inhaler technique training, care linkage, and follow-up calls	No CHW program	Yes	Intervention study with historical control (50 patients)	6 months	13
Enard KR 2013^51^	Texas	Patients who visited the ED for primary care-related reasons at Memorial Hermann Health System	–	ED-based patient navigation program (Telephone calls for ED-based patient navigation by bilingual CHWs providing in-person barrier assessment, linkage to medical homes, primary care education, and follow-up telephone calls within 3–10 days)	Usual care	Yes	Quasi-experimental (I: 1905; C: 11,737 patients)	12 months	12
Li Y 2017^52^	Texas	Female 18 years or older enrolled in Care Link	Urban Hispanic women	Telephone calls (Telephone-based diabetes self-management support by CHWs with culturally tailored education, goal setting, and care coordination)	Current practice	Yes	Microsimulation modeling approach (4500 patients)	36 months	14
Fiori KP 2024^53^	New York	Patients with Health-related social needs	–	CHWI Program (Bridge between social and clinical care by addressing health needs and improving access to healthcare for disadvantaged populations)	Usual care	Yes	Pre-post Intervention (I: 1245; C: 3175 patients)^82^	12 months	8
Mechanic OJ 2022^54^	Massachusetts	heterogeneous, tech-naïve primary care cohort with disparities in access	Tech-Naïve population with disparities in access	Telehealth Patient Navigator Program (Telephone calls to improve the access)	Usual care without a navigator	Yes	Quasi-experimental design (I: 1035; C: 3031 patients)	3 months	14
Lawlor MS 2013^55^	North Carolina	Patients with diabetes	–	Healthy Living Partnerships to Prevent Diabetes (In-person visiting & telephone calls by CHWs for patient education, follow-up support, and linkage to healthcare services)	Usual care (visits with a registered dietitian and monthly newsletters)	No	RCT (I: 151; C: 150 patients)^83^	24 months	10
Herman PM 2022^56^	Arizona	50 years of age or older, English or Spanish speaking, and were out of compliance for CRC screening guidelines at that time, either never screened or currently due	Out of compliance population and underserved population	Community-to-clinic navigation Program (Telephone calls, clinic appointment by CHWs for patient follow-up, education, and care coordination)	Community-based group education	No	RCT (I: 211 patients; C: 134patients)^84^	36 months	12
Garson A Jr 2012^57^	Texas	Texas: Children under 19 years old visit a federally qualified health center	–	Grand-Aides® Program (Home visits by CHWs providing health assessment, tailored health education, and linkage to healthcare and community services)	Usual care without Grand-Aides	Yes	Texas: Prospective, cross-sectional study (457 patients)	Texas: 1 month	12
		Virginia: Medicaid-insured children under 19 visiting an ED					Virginia: Prospective, cross-sectional study (402 patients)	Virginia: 15 months	
Galbraith AA 2017^58^	Massachusetts	High-risk medical service patients		Hospital visits & Telephone outreach weekly by PNs for patient education, follow-up support, and care coordination	Usual care	Yes	RCT (I: 448 patients; C: 527 patients)^85^	6 months	12
Corliss J 2015^59^	Alabama	Primarily Medicare patients within the UAB network	–	LPN program (One-one-one phone calls & In-person support by LPN providing treatment education, symptom recognition guidance, care coordination, and end-of-life care discussions)	Usual care without a navigator	Yes	Quasi-experimental (30,589 patients)	24 months	11
Lightner JS 2025^60^	Kansas	Patients who are 18 years old or older living with HIV	Unemployed or under-employed patients	Improve housing and employment for PWH	Usual care without a navigator	Yes	Ambispective interventional study (195 patients)^86^	24 months	11
Fawcett KJ Jr 2018^61^	Michigan	Adults diagnosed with diabetes and/or congestive heart failure	Underserved rural community	Core Health Program (Home visits by CHWs providing health education, linkage to services, barrier assessment, and follow-up support)	No CHW program	Yes	Quasi-experimental (277 patients)	12 months	12
Christiansen E 2017^62^	Nevada	Super-utilizers who are having three or more ED visits in six months	–	HPN Program (Home visits and follow-up phone calls by CHWs for cardiovascular risk screening, lifestyle counselling, goal setting, and referral to healthcare and community resources)	No CHW program	Yes	Intervention study (1437 HPN members)	3 months	14
McCarthy D 2020^63^	Ohio	Chronically ill adult patients including diabetes, hypertension	–	Pathways to Health Program (Monthly home education visits and care coordination by CHWs for adults with chronic conditions, including social and medical needs assessment, barrier reduction, goal setting, and linkage to community services)	No CHW program	Yes	Intervention study (180 patients)	12 months	15
Gillam P 2020^21^	South Carolina	Patients who were uninsured with at least one chronic disease or behavioral health issue and participants that have social health needs	–	Access Health Spartanburg Program (Office visits, phone calls, home visits and resource referrals to address needs related to housing, transportation, behavioral health, food security, vision, and medication access)	No CHW program	NR	Pre-post intervention study (I: 75; C: 417)	6 months	15
		Pregnant mothers with low incomes up to 24 years of age	Low-income patients with limited access	Birth Matters Program (Home visits, access to health services, housing, mental health, intimate partner violence, and health equity based on racial disparities of infant/maternal mortality)	No CHW program	NR	Pre-post intervention study (I: 79; C: 79)	6 months	15
		Patients who were low-income and uninsured and were enrolled in the Access Health programs in the Upstate region	Low-income patients with limited access	Prisma Health Upstate Program (Phone calls, transportation arrangement, patient education, social support, discussion of community resources, identifying access to food, providing utility assistance and health care services)	No CHW program	NR	Pre-post intervention study (I: 148 patients)	30 months	15
		Population that was dually eligible for Medicare/Medicaid with uncontrolled hypertension/diabetes, or multiple ED visits within the past 12 months	–	Tandem Health Program (Phone calls, office visits, Referrals for food security, housing, transportation and health education)	No CHW program	NR	Pre-post intervention study (I: 41 patients)	18 months	15

AAHP: Asthma and Healthy Homes Program; BTH: Bridges to Home; CCMN: Colorectal Cancer Male Navigation; CHW: Community health workers; CHWI: Community Health Worker Institute; COACH: Community Outreach and Cardiovascular Health; CVD: Cardiovascular disease; DCAS: Diabetes Care in American Samoa; ED: Emergency department; HIV: Human-immune deficiency virus; HPN: Health Plan of Nevada; IMPaCT: Individualized Management for Patient-Centered Targets; ITT: Intention to treat; LHW; Lay Health Worker; LPN: Lay patient navigator; MHI: Men's Health Initiative; MIND: Maximizing Independence; NR: Not-reported; PTP: Prenatal Technology Platform; PWH: Patients with HIV; READY: Reducing Ethnic/Racial Asthma Disparities in Youth; UTCO: University of Texas Community Outreach.

### CHW programme outcomes

Across analyses, CHW interventions consistently yielded positive clinical outcomes including reduced healthcare utilization (hospital, ED, urgent care and pharmacy visits; n = 37; 90.2%),^12^^,^^19^, ^20^, ^21^^,^^34^^,^^35^^,^^37^, ^38^, ^39^, ^40^, ^41^, ^42^, ^43^, ^44^, ^45^, ^46^, ^47^, ^48^, ^49^, ^50^, ^51^, ^52^^,^^54^, ^55^, ^56^, ^57^, ^58^, ^59^^,^^61^, ^62^, ^63^, ^64^ improved clinical endpoints (HbA1C, BP, QALY, symptom-free days) (n = 30; 73.2%),^12^^,^^19^, ^20^, ^21^^,^^34^^,^^36^^,^^37^^,^^39^, ^40^, ^41^, ^42^, ^43^, ^44^, ^45^^,^^47^, ^48^, ^49^, ^50^^,^^52^^,^^55^^,^^60^^,^^61^^,^^64^ reduced productivity loss (n = 8; 19.5%),^34^^,^^42^^,^^47^^,^^49^^,^^55^ and improved hospital and insurance access (n = 7; 17.1%).^37^^,^^39^^,^^41^^,^^43^^,^^54^^,^^56^^,^^57^ Outcomes were measured using pre-post intervention studies (n = 19; 46.3%),^19^^,^^20^^,^^34^^,^^35^^,^^37^^,^^38^^,^^40^^,^^42^^,^^46^^,^^48^^,^^53^^,^^60^^,^^61^ comparative non-randomized experimental studies (n = 10; 24.4%),^36^^,^^39^^,^^41^^,^^45^^,^^50^^,^^51^^,^^54^^,^^59^^,^^62^^,^^63^ randomized controlled trials (n = 7; 17.1%),^12^^,^^43^^,^^49^^,^^55^^,^^56^^,^^58^^,^^64^ and observational studies (n = 4; 9.8%)^44^^,^^47^^,^^52^^,^^57^ with sample sizes ranging from dozens^46^ to tens of thousands^40^^,^^58^ (Tables 1 and 2).

### Community and patient engagement

Strong patient and community engagement was consistent across the included CHW intervention studies. Twenty analyses (n = 13; 48.8%)^12^^,^^20^^,^^34^^,^^36^^,^^41^^,^^43^^,^^46^^,^^47^^,^^49^^,^^50^^,^^53^^,^^55^^,^^58^^,^^60^^,^^61^ explicitly demonstrated or reported stakeholder and community engagement during the design, planning, implementation, and execution. Engagement strategies include patient interview or validated questionnaire assessment to understand needs (n = 3; 7.3%),^12^^,^^20^^,^^43^ healthcare stakeholder interview to plan CHW programmes (n = 1; 2.4%),^43^ culturally matched CHWs as trusted community representative (n = 7; 17.7.%)^34^^,^^36^^,^^49^^,^^50^^,^^55^^,^^58^^,^^61^ academic-community research partnerships with rural community advisory boards (n = 3; 7.3%),^41^^,^^46^^,^^53^ interdepartmental collaborations (n = 2; 5%),^43^^,^^60^ and local CHW organizations and network collaborations (n = 4; 9.7).^46^^,^^47^^,^^53^^,^^60^ Community engagement details are provided in Table 1.

### Characteristics of ROI evaluation

#### Perspective and time-horizon

The healthcare system perspective (n = 30; 73.17%)^20^^,^^21^^,^^34^^,^^36^^,^^38^, ^39^, ^40^, ^41^^,^^43^^,^^45^^,^^46^^,^^48^^,^^51^^,^^53^, ^54^, ^55^, ^56^, ^57^, ^58^, ^59^, ^60^, ^61^, ^62^, ^63^ was predominantly adopted as most CHW interventions were healthcare institution-sponsored programmes. Payer and societal perspectives were considered in 16 (39%)^12^^,^^19^^,^^21^^,^^34^^,^^35^^,^^37^^,^^38^^,^^44^^,^^50^^,^^64^ and 8 (19.5%)^34^^,^^36^^,^^42^^,^^46^^,^^47^^,^^49^^,^^52^^,^^55^^,^^60^ analyses, respectively. Time horizon varied from 3 months^54^ to over 20 years^47^ (Tables 1 and 3).

**Table 3:** The characteristics and components of ROI calculation.

Author, year	Components of the cost of intervention	Perspective	Assumptions on case load	Economic modelling used	Discount rate	Time horizon	Total cost of intervention	Total cost saving	ROI value
Kangovi S 2020^12^	Personal, equipment, services, office space rent, and Indirect cost	Payer	330 patients/6 CHWs/year	Simple cost analysis	NR	1 year	$567,951	$1,401,308	$2.47
London K 2017^34^	Salary, Fringe, travel, office supplies, equipment, training, and supervision cost	Healthcare system, Payer and Societal	96/CHW/year	Simple cost analysis	3.3%	3 years	$229,000	$427,000	$1.86
	Salary, Fringe, travel, office supplies, equipment, training, and supervision cost	Healthcare system, Payer and Societal	79/CHW/1.5 year or 158/cohort	Simple cost analysis	3.3%	3 years	$435,000	$487,200	$1.12
	Salary, Fringe, travel, office supplies, equipment, training, and supervision cost	Healthcare system, Payer and Societal	36/CHW/year or 72/cohort	Simple cost analysis	3.3%	3 years	$944,000	$2,265,600	$2.40
	Salary, Fringe, travel, office supplies, equipment, training, and supervision cost	Healthcare system, Payer and Societal	148/CHW/year	Simple cost analysis	3.3%	3 years	$194,000	$388,000	$2.00
Willink A 2020^35^	Salary and Fringe for Phone calls, messages, e-mails, in-person visit and email	Payer	NR	Simple cost analysis	NR	5 years	$2,257,200	$2,528,064	$1.12
Marshall ET 2020^19^	NR	Payer	NR	Simple cost analysis	NR	1 year	$38,731	$51,900	Completed all visits: $0.49; High end-users: $1.34
Ryabov I 2014^36^	Program personnel cost	Healthcare system and Societal	NR	Markov and Monte-Carlo simulation	3%	2 years	$15,362	$27,620	$1.80
van der Goes DN 2019^37^	Annual maintenance and operation costs and human capital/indirect costs	Payer	NR	Simple cost analysis	3%	10 years	$9,555,226	$11,332,899	$1.19
Whitley EM 2006^38^	Personnel cost, copayment, medical supplies and travel	Healthcare system and Payer	NR	Simple cost analysis	NR	1.5 years	$112,129	$255,655	$2.28
Wilson FA 2015^39^	Personnel cost, colonoscopy cost, cancer treatment cost	Healthcare system	NR	Markov Modelling	3%	1 year	$185,357	$424,760	$2.29
Patel MI 2020^40^	Healthcare utilization cost between Intervention and comparator group	Healthcare system	NR	Simple cost analysis	NR	1 year	$765,000	$3,080,400^a^	$4.03^a^
Cramer ME 2018^41^	Mobile technology services, CHW training and program management	Healthcare system	NR	Simple cost analysis	NR	15 months	$154,519	$325,335	$1.90
Cardarelli R 2018^20^	CHW salary and the operational cost	Healthcare system	NR	Simple cost analysis	NR	1 year	$39,936	Non-ACO, DRG-only: −$26,846; Non-ACO, with P4P Contracts: 280,563 ACO Model: 1,544,574	Non-ACO, DRG-only: $−0.67; non-ACO, with P4P Contracts: $7.03; ACO Model: 38.68
Naufal G 2022^42^	CHW salaries, and material costs	Societal	NR	Simple cost analysis	NR	3 years	$86,655	$1,371,612	$15.8
Morgan AU 2016^43^	CHW salaries and supplies	Healthcare system	NR	Simple cost analysis	NR	1 year	Pilot: $65,000 RCT: $60,000	$225,000	2013: $1.80; 2016: $2.00
Bhaumik U 2020^44^	Salary, Fringe, supplies cost, mobile phone, tablet for data collection and travel	Payer	NR	Simple cost analysis	NR	5 years	$78,750	$107,888	$1.37
Vohra AS 2020^45^	Salary, benefits, supply cost and indirect cost	Healthcare system	12.8 patients/CHW/year	Markov modelling	NR	1 year	$128,115	$128,324	$1.00
Turcotte DA 2014^46^	Healthcare utilization cost between Intervention and comparator group	Healthcare system and Societal	NR	Simple cost analysis	NR	1 year	$32,640	4 weeks: 38,522; 6 months: 394,332; 1 year $821,304	4 weeks: 1.18; 6 months: 12.08; 1 year: 25.16^a^
Brown HS 3rd, 2012^47^	Personnel cost, supplies, travel and indirect cost	Societal	NR	Simple cost analysis	3%	20 years	$5,494,185	$22,251,449	$4.05
Ohuabunwa U 2021^48^	Salary, and training	Healthcare system	NR	Simple cost analysis	NR	1 year	$350,000	$7,512,883^a^	$21.40^a^
Campbell JD 2015^64^	Salaries, benefits, administrative, logistical, supplies and training cost	Payer	NR	Simple cost analysis	NR	1 year	$108,878	$206,514^a^	$1.90
Huang SJ 2019^49^	Staff cost and resource utilization cost including indirect cost	Societal	NR	Simple cost analysis	NR	2 years	$70,453	$213,473^a^	$3.03^a^
Margellos-Anast H 2012^50^	Salaries and supplies	Payer	100 patients/CHW/year	Simple cost analysis	NR	1 year	$22,953	$128,080^a^	$5.58
Enard KR 2013^51^	Healthcare utilization cost between Intervention and comparator group	Healthcare system	NR	Simple cost analysis	NR	1 year	$45,880	$504,680	$11
Li Y 2017^52^	Staff salaries, health promotion, media, outreach cost and test cost	Societal	NR	Markov modelling	3%	3 years	$1,399,815 over 3 years	$12,360,367^a^	$8.83
Fiori KP 2024^53^	Salary, benefits, and maintenance cost	Healthcare system	NR	Simple cost analysis	NR	1 year	$515,992	$1,274,501	$2.47
Mechanic OJ 2022^54^	Salary, facility cost and reimbursement	Healthcare system	NR	Simple cost analysis	NR	3 months	$17,878	$29,265	$1.64
Lawlor MS 2013^55^	Intervention delivery cost, and cost for time spend during the intervention (indirect cost)	Healthcare system and Societal	NR	Simple cost analysis	NR	2 years	$127,500	$283,050	$2.22
Herman PM 2022^56^	Salary, travel, lab test, event fee, materials, supplies, laptop, equipment, food, parking and recruiting cost	Healthcare system	NR	Simple cost analysis	NR	3 years	$57,181	$108,644	$1.90
Garson A Jr 2012^57^	Salary, Fringe benefits, technology, transportation, and training	Healthcare system and Payer	200–250 families/CHW/year	Simple cost analysis	NR	15 months	$40,503	Texas: $51,789; Virginia: $91,008	Texas: 1.27^a^; Virginia: 2.25^a^
Galbraith AA 2017^58^	Salary, and telephone outreach cost	Healthcare system	NR	Simple cost analysis	NR	6 months	$216,384	∼$642,000	$2.97
Corliss J 2015^59^	Salary, phone, and training	Healthcare system	30% of the Medicare patients/40 LN	Simple cost analysis	NR	2 years	$15,000,000 over 2 years	$236,100,000 over 2 years	$15.7
Lightner JS 2025^60^	Salary, Fringe, travel, HER fee, consulting, training, telecommunication, and indirect cost	Healthcare system and Societal	NR	Simple cost analysis	NR	3 years	$1,364,828	$18,377,317	$13.50
Fawcett KJ Jr 2018^61^	Healthcare utilization cost between Intervention and comparator group	Healthcare system	NR	Simple cost analysis	NR	1 year	$552,636	$757,111	$1.37
Christiansen E 2017^62^	Healthcare utilization cost between Intervention and comparator group	Healthcare system	NR	Simple cost analysis	NR	14 months	$278,331	$503,384	$1.80
McCarthy D 2020^63^	Healthcare utilization cost between Intervention and comparator group	Healthcare system	NR	Simple cost analysis	NR	1 year	$1,440,720	$2,478,038	$1.72
Gillam P 2020^21^	Salary and benefits, percentage of supervisors' salary and benefits, mileage reimbursement, cell phone allowance, costs for work phones and computers, CHW training, and marketing for the CHW program	Healthcare system and Payer	NR	Simple cost analysis	NR	1 year	$79500	$772,725^a^	$9.72
		Healthcare system and Payer	NR	Simple cost analysis	NR	1 year	$156,668	$332754^a^	$2.12
		Healthcare system and Payer	NR	Simple cost analysis	NR	1 year	$551,983	$3,400,000^a^	$6.17
	Healthcare utilization cost between Intervention and comparator group	Healthcare system and Payer	NR	Simple cost analysis	NR	1 year	$140,044	$92 865^a^	$0.66

CHW: Community health worker; NR: Not reported.

a Calculated by authors using the available data.

#### Model used for ROI analysis

The majority of analyses (n = 37; 87.9%)^12^^,^^19^, ^20^, ^21^^,^^34^^,^^35^^,^^37^^,^^38^^,^^40^, ^41^, ^42^, ^43^, ^44^^,^^46^, ^47^, ^48^, ^49^, ^50^, ^51^^,^^53^, ^54^, ^55^, ^56^, ^57^, ^58^, ^59^, ^60^, ^61^, ^62^, ^63^, ^64^ used simple economic evaluations with cost comparison analyses. All studies used usual or standard care without a CHW intervention as the comparator, with evaluations conducted either through pre-post analyses or comparisons with a concurrent control group. The primary ROI calculating method consistently used across studies was the ratio of total cost savings to total intervention cost, although parameters and inputs varied when calculating cost components. Markov models were used in 4 analyses (9.7%),^36^^,^^39^^,^^45^^,^^52^ and Monte-Carlo simulation in one analysis (2.4%).^36^ Discount rates were explicitly reported in 9 analyses (24.4%)^34^^,^^36^^,^^37^^,^^39^^,^^47^^,^^52^ (Tables 1 and 3).

#### Cost-considerations

Most analyses (n = 40; 97.6%)^12^^,^^19^, ^20^, ^21^^,^^34^, ^35^, ^36^, ^37^, ^38^, ^39^, ^40^, ^41^, ^42^, ^43^, ^44^, ^45^^,^^47^, ^48^, ^49^, ^50^, ^51^, ^52^, ^53^, ^54^, ^55^, ^56^, ^57^, ^58^, ^59^, ^60^, ^61^, ^62^, ^63^, ^64^ considered personnel costs (salaries, fringe benefits) as major inputs, and 32 analyses (78%)^12^^,^^19^^,^^21^^,^^34^, ^35^, ^36^^,^^39^, ^40^, ^41^, ^42^, ^43^^,^^45^^,^^47^^,^^48^^,^^50^^,^^51^^,^^53^, ^54^, ^55^^,^^57^^,^^59^, ^60^, ^61^, ^62^, ^63^, ^64^ considered CHW training costs when calculating the intervention cost. Some studies also considered patient time during intervention and patient co-payment^38^^,^^55^ when calculating the personnel cost. Non-personnel costs (equipment, services, office space rent) and indirect costs were considered by 30 (73.2%)^12^^,^^19^^,^^21^^,^^34^, ^35^, ^36^, ^37^, ^38^, ^39^, ^40^, ^41^, ^42^^,^^44^^,^^46^^,^^47^^,^^49^^,^^52^^,^^55^, ^56^, ^57^, ^58^^,^^60^^,^^62^^,^^64^ and 7 (17%)^12^^,^^37^^,^^45^^,^^47^^,^^49^^,^^55^^,^^60^ analyses, respectively (Tables 1 and 3).

#### ROI values

Inflation-adjusted median annual CHW programme intervention cost was $155,275 (IQR: $61,800–$137,610), ranging from $10,177^36^ to $10.2 million.^59^ Median annual cost-saving was $403,298 (IQR: $137,610–$1,274,501), ranging from $18,298^36^ to $160.5 million.^59^ Median ROI was $2.12 (IQR: 1.64–4.03) per dollar spent, with an average of $4.11 (SD: 4.81). Median ROI value was higher among studies with societal ($3.54; IQR: 2.57–5.25) compared with payer ($2.12; IQR: 1.55–5.87) and healthcare system ($1.95; IQR: 1.68–2.37) perspectives. The region-specific analysis indicates that the median ROI was higher among the studies from the Southern region ($3.17; IQR: 2.04–10.04) compared with the Midwest ($1.91; IQR: 1.46–4.71), West ($1.90; IQR: 1.85–2.65), and Northeast ($1.83; IQR: 1.36–2.42) regions. Author-reported intervention cost, net saving, and ROI value are presented in Table 3, with detailed annual conversion and inflation adjustment calculation in Supplementary File S5.

#### Factors affecting the ROI

The median ROI did not differ significantly by the perspective (p = 0.598), region (p = 0.260), target population (p = 0.378), programme duration (p = 0.940) or inclusion of sensitivity analyses (p = 0.491). The results are presented in Supplementary File S6. Although studies that included a specific or combined CHW role generally observed a higher ROI than those that did not, only programmes that incorporated the provision of direct services demonstrated a significant difference (p = 0.040) in ROI (Supplementary File S7).

#### Robustness of the model

Seventeen studies (48.5%)^12^^,^^19^^,^^20^^,^^35^^,^^36^^,^^39^^,^^41^, ^42^, ^43^^,^^47^^,^^49^^,^^52^^,^^54^^,^^56^^,^^57^^,^^63^^,^^64^ conducted sensitivity or scenario analysis. Among these, 11 (31.4%)^20^^,^^35^^,^^36^^,^^39^^,^^41^^,^^42^^,^^47^^,^^49^^,^^54^^,^^63^^,^^64^ performed one-way, 3 (8.5%)^12^^,^^39^^,^^52^ performed two-way, and 2 (5.7%)^36^^,^^56^ performed probabilistic sensitivity analysis. Five studies (14.3%)^19^^,^^41^^,^^43^^,^^57^^,^^64^ performed scenario analysis (Fig. 3). Analyses varied intervention cost,^42^^,^^52^^,^^54^^,^^64^ intervention cost components,^54^^,^^64^ effectiveness measures,^12^^,^^39^^,^^42^^,^^47^^,^^49^ target population group,^19^ discount rates,^47^ case load or admissions number,^12^ or payment system.^20^^,^^35^ One study removed outlier patient data^41^ to check the robustness, and two studies^37^^,^^43^ performed year-wise ROI. Varying the target sub-population group (all vs higher-use patients),^19^ programme effectiveness (increase the %),^39^^,^^47^^,^^52^ discount rate (decrease to 0%),^39^^,^^47^ longer programme duration (2 year and 5-year benefits),^35^^,^^37^^,^^43^ median spending growth (11.9% faster per quarter),^35^ increased access to the programme,^36^ more case load or admissions number,^12^ lowering the intervention cost,^42^^,^^52^^,^^54^^,^^64^ and different payment/reimbursement system (payment for broad range of services),^20^^,^^35^ were associated with higher ROI. Whereas, varying the effectiveness (decreasing the %),^39^^,^^47^ discount rate (increasing to 6%),^39^^,^^47^ increasing the intervention cost,^42^^,^^52^^,^^54^^,^^64^ and different payment system (fixed payment for specific services)^20^ were associated with lower ROI.

### Equity concern and distributional effects

No studies, except Wilson et al.^39^ discussed equity considerations in ROI analyses through targeting the interventions to Hispanic men, a population that often experience health disparity. Though the Wilson study incorporated both ethnic and gender dimensions of the intervention, it did not perform a formal comparative equity analysis. Thirteen studies (37.1%)^21^^,^^34^^,^^36^^,^^38^^,^^39^^,^^41^, ^42^, ^43^^,^^45^^,^^47^^,^^50^^,^^52^^,^^61^ considered equity while implementing CHW programmes by serving specialized or marginalized populations, but did not estimate distributional effects. A lack of equity-focused analysis in ROI evaluations reduces findings’ relevance for addressing health disparities and informing inclusive policy decisions.

## Discussion

CHW interventions in the US consistently demonstrate positive ROI, underscoring the financial viability and significant value these programmes have in enhancing population health.^12^^,^^40^^,^^66^ These frontline public health workers serve as crucial bridges between underserved populations and the healthcare system, providing multifaceted services.^1^^,^^14^^,^^87^ Their roles commonly include health education, patient navigation, care coordination, and social support among vulnerable individuals and those with chronic health conditions.^1^^,^^14^^,^^39^^,^^40^^,^^51^^,^^87^ Widespread positive clinical outcomes include fewer hospital and emergency department visits, improved disease management, enhanced healthcare access, and increased preventive screening rates.^39^^,^^56^^,^^88^^,^^89^ Unlike prior reviews that focused mainly on health outcomes or cost-effectiveness without consolidating ROI evidence,^22^^,^^23^ this analysis offers the most comprehensive synthesis of US-based CHW ROI evaluations to date.

We harmonized the cost estimates and ROI to 2024 USD and applied structured frameworks for CHW roles and quality appraisal. We found a median ROI of $2.12 (IQR: 1.64–4.03) per dollar invested, which aligns with the previous works by Masters et al., where they recorded a median ROI of 2.2 (range 0.7–6.2) for health promotion interventions.^17^ This pattern was consistent with real-world evidence from well-organized CHW interventions, which reported an ROI between $2 and $2.50 per dollar invested.^12^^,^^21^^,^^34^^,^^38^^,^^39^^,^^43^^,^^53^^,^^55^^,^^57^^,^^64^ A previous systematic review reported a slightly higher ROI for local public health interventions, and our analysis similarly observed a higher ROI for studies adopting a societal perspective ($4.10 vs $3.54).^17^

The CHW ROI studies were more concentrated in Southern and Northeastern states, potentially due to better funding opportunities, and institutional supports for CHW activities in the region.^25^^,^^90^ Texas and Massachusetts were well represented, and both states have established CHW funding mechanism laws, CHW Medicaid provisions, or other public assistance legislation.^24^ Notably, region-specific analysis showed that the Southern region achieved a higher median ROI than other regions, suggesting that geographic and policy contexts along with other factors may influence financial returns. We conducted multiple analyses of CHW roles, both individually and in combination, to identify services that might generate higher returns and inform future program development. Only CHW programmes that incorporated the provision of direct services demonstrated a significantly higher ROI. These findings suggest that integrating direct services such as administering basic first aid, conducting screening tests and addressing basic needs of the people may be an important component for future CHW programmes to achieve a higher ROI while enhancing community benefits. Further granular analyses with state-, region-, or territory-level cost input and CHW services data would help to explore this in future studies.

Sustainable financing remains a persistent challenge for CHW programs despite the evidence on positive ROI, as states continue to have a non-uniform funding approach. Funding instability and lack of consolidated cost data further complicate the ROI assessment across settings,^91^ which underscore the real impact. Medicaid has emerged as one potential pathway to support CHW reimbursement and improve access to CHW services. According to recent National Academy for State Health Policy (NASHP) reports, six states initially had state plan amendments (SPAs) authorizing Medicaid reimbursement for CHW services. As of December 2023, 15 states had approved SPAs, with several others indicating that SPAs in development or legislative mandates for future introduction.^92^ Additionally, CHW services often depend on limited philanthropic and institutional funding, making it difficult to meet growing organizational and population demands,^1^^,^^63^ which needs to be strengthened with sustainable supports and jointly planned financial contracts with relevant organizations.

Programme costs typically range from tens of thousands to millions of dollars, with overwhelmingly positive ROI figures up to $38.68,^20^ and net savings often reaching millions. Despite this consistent positive trend, Cardarelli et al.^20^ reported negative ROI of −$0.67 for programmes that runs under a fixed payment per hospitalization episode related to diagnosis (Non-ACO, DRG-only payment models). In contrast, the study showed significant gains and good ROI with other alternative payment structures, including pay-for-performance, that reward quality outcomes (non-ACO with P4P Contracts model) and the ACO models that incentivize cost-savings and coordinated care. This indicates that payment structures or specific contextual scenarios significantly influence financial outcomes rather than general CHW interventions ineffectiveness. ROI variation and CHW intervention impact are influenced by factors including specific disease addressed, intervention intensity, and assumed CHW caseload.^87^^,^^93^, ^94^, ^95^

We found a few studies that included distributional effects or equity considerations in their ROI analyses, though some studies incorporated equity during CHW programme implementation by targeting specialized populations. Considering equity is crucial as CHW programmes often serve marginalized groups. Overlooking equity in ROI may mask health outcomes disparities and resource allocation inequities, ultimately undermining health equity goals.^87^^,^^96^ This perpetuates health access barriers and challenges, particularly among lower socioeconomic groups.^97^ Since CHW programs vary in their purpose and are not always clinical in nature, engaging representatives from end users in the design and implementation of the programme is very important. These include patients, community members, and CHWs themselves, whose participation helps to ensure that programmes are relevant, feasible, and culturally appropriate to meet community's need.^1^^,^^43^^,^^98^ Such engagement also helps to identify barriers, refine messaging, improve accessibility, and foster trust within large populations. Embedding end-user perspectives into programme development can strengthen equity and sustainability, which further reinforce the economic and public health value of the programmes.^16^^,^^43^^,^^99^, ^100^, ^101^, ^102^

Most studies adopted healthcare system and payer perspectives without performing affordability analysis by integrating potential CHW programme remuneration through government initiatives or medical reimbursement systems. Future analyses should adopt a more affordability-focused approach involving government and community partners to capture broader population data and potential renumeration-based analyses, enhancing policy relevance.^23^ Key messages to stakeholders, including policymakers and healthcare administrators, are that CHW interventions consistently demonstrate positive ROI and represent financially sound public health investment.^4^^,^^12^^,^^17^ Our findings can provide valuable information for policy and funding decisions. By understanding economic value, stakeholders can maximize CHW programmes’ societal and economic benefits, strategically allocating resources to implement and sustain community-based initiatives, improving population health outcomes, and reducing healthcare costs.^14^^,^^90^^,^^103^^,^^104^

### Limitations

This review acknowledges several limitations and areas of potential improvement. Primary drawbacks include the methodological heterogeneity in intervention and cost components, target population, duration of intervention, inconsistent comparisons, outcome components and measurement, and time horizons. Hence, we did not perform a meta-analysis. However, a dedicated meta-analytic study would be beneficial in the future. Some studies incorporate societal costs like productivity gains, while others focus solely on healthcare system savings. Publication bias is a concern since studies with positive ROI results are more likely to be published, potentially overestimating true economic benefits. For instance, four studies^21^^,^^34^^,^^62^^,^^63^ accounting for 10 (24.3%) of 41 analyses were not peer-reviewed publications but were, instead, organizational reports. More unpublished reports might exist. Although some studies met the overall quality metrics, they were missing critical cost data or cost components or failed to apply necessary cost adjustments, which may further limit the precision and comparability of ROI estimates. Another major concern is short-term ROI analysis (e.g., one year), potentially underestimating substantial long-term economic benefits, especially for preventive care and chronic disease management programmes. Despite inflation adjustment, state-level variation in cost of living may influence ROI estimates, as we observed a high ROI in the Southern region. However, limited reporting of region or territory-specific local cost inputs prevented further granular analyses, highlighting an area for future research. Finally, the US-only scope of this review limits generalizability to other countries or healthcare systems. Additionally, clinical and methodological heterogeneity in component selection, ROI modelling, and possible publication bias should be acknowledged.

### Future directions

To improve future evaluations and enhance the evidence base, strategies should focus on standardizing ROI methodologies. Future studies should ensure comprehensive data collection including healthcare system and societal costs, with subgroup and sensitivity analyses to enhance the generalizability of the findings. More equity-focused research would provide a better understanding of CHW value. Conducting longer-term economic evaluations is crucial to fully capturing CHW interventions’ sustained benefits and cost savings, particularly for chronic conditions. Addressing publication bias through diligent grey literature searches and encouraging null or negative findings reporting would strengthen evidence.

Finally, additional studies targeting specific gaps could produce insights and lessons that could potentially encourage CHW implementation by various stakeholders, including state and federal governments. Because few existing studies address affordability, future government-initiated research studies incorporating actual societal data should be conducted to understand the real-world CHW programme affordability. Medicaid and other health policies generally prohibit paying for non-traditional or health-related services.^25^^,^^63^ Recent state policies are expanding Medicaid CHW services coverage,^105^ and more affordability-based analyses could make the case for reimbursement for CHW services. Including patient and cultural representatives ensures CHW programmes are clinically relevant and culturally meaningful. Their input improves accessibility, trust, and effectiveness by identifying barriers, refining communication, and guiding equitable strategy development.^1^^,^^43^ Future studies should incorporate more social return on investment (SROI)-based analyses capturing CHW interventions’ broader social and economic benefits. SROI provides more comprehensive frameworks incorporating social, health, and economic outcomes for evaluating long-term value beyond direct cost savings. These evaluations can include community engagement, education levels, physical and mental health, care access, employment rates, indirect costs, wage loss, and socioeconomic levels for more holistic, policy-relevant estimation.^106^, ^107^, ^108^, ^109^

### Conclusion

In this systematic review, we found that CHW interventions consistently showed high ROI and substantial net cost savings across studies. However, significant methodological and contextual heterogeneities at CHW, system, analysis, and implementation levels influence key outcomes and limit generalizability. This review synthesizes the ROI evidence across diverse settings and contexts to provide a comprehensive picture. Additionally, this review highlights the gaps in the equity consideration and methodological consistency to guide future research. To enhance the reliability and policy relevance of future evaluations, standardized frameworks identifying common indicators, model inputs, and evaluating SROI should be developed. Such frameworks would support more consistent, transparent assessments, enabling policymakers and research funders to make informed resource allocation and scaling of CHW programme scaling decisions.

## Contributors

Conceptualisation and study design: Nathorn Chaiyakunapruk (NC), Muhammed Rashid (MR), Jeong-Yeon Cho (JC), Surasak Saokaew (SS); Search strategy development: MR, NC, JC; Study screening and selection: Max Fu (MF), Pattranit Jitareewong (PJ), MR, SS; Data extraction: MF, PJ, MR; Quality assessment: MF, PJ, MR; Data analysis and interpretation: MR, NC, SS; Drafting of the manuscript - Writing: MR, MF, SS; Drafting of the manuscript – Revising: MR, MF, SS, JC, Richard E. Nelson (RN), Rachel M. Ceballos (RC), NC; Guarantor: SS, NC; Funding acquisition: None.

## Data sharing statement

The study protocol is publicly available at PROSPERO, CRD42023456789. The data used for this systematic review will be made available on request to the corresponding author.

## Editor note

The Lancet Group takes a neutral position with respect to territorial claims in published maps and institutional affiliations.

## Use of artificial intelligence statement

The authors used artificial intelligence tools including DeepSeek and Grammarly to assist with language refinement and editing of the manuscript (improving grammar, sentence structure, and manuscript readability). We confirm that all AI-assisted processes were critically reviewed by the authors to ensure the integrity and reliability of the results. The final decisions and interpretations presented in this article were solely made by the authors.

## Declaration of interests

No competing interests to declare.
