# Supplementary material for: Return on investment of community health workers in the United States: a systematic review
Source: Lancet Reg Health Am. 2026 Apr 21;58:101469. doi: 10.1016/j.lana.2026.101469 (PMC13123591; doi:10.1016/j.lana.2026.101469)
Supplement: Supplementary Files [file mmc1.docx]

**Table of Contents**

[Supplementary file S1: The detailed search Strategy 2](#_Toc224554075)

[S1A: PubMed 2](#_Toc224554076)

[S1B. Embase 3](#_Toc224554077)

[S1C: Web of Science 4](#_Toc224554078)

[S1D: EconLit (Via EBSCOhost) 5](#_Toc224554079)

[S1E: CEA Registry by Tufts 6](#_Toc224554080)

[Supplementary file S2: Detailed description of CHW activities organized under the seven major role categories. 7](#_Toc224554081)

[Supplementary file S3: List of excluded studies (N=69) 8](#_Toc224554082)

[Supplementary file S4: The quality assessment of included studies 16](#_Toc224554083)

[Supplementary file S5: The intervention cost, total net cost-saving and the ROI 20](#_Toc224554084)

[Supplementary file S6: Factors affecting the ROI 23](#_Toc224554085)

[Supplementary file S7: Association between CHW roles and ROI 24](#_Toc224554086)

# Supplementary file S1: The detailed search Strategy

## S1A: PubMed

| **No.** | **Search strategy** | **No. of Hits** |
| --- | --- | --- |
| Domain 1: Community Health Worker | | |
| #1 | “Community Health Worker”[Mesh] OR “Community Health Worker”[Title/Abstract] OR “Community Health Workers” OR “Community Health Work*” OR “Community Health Aide*” OR “Village Health Worker*” OR “village-based family planning worker”[Title/Abstract] OR “lay health*” OR “lay health worker”[Title/Abstract] OR “health coach”[Title/Abstract] OR “health aide” [Title/Abstract] OR “health educator”[Title/Abstract] OR “patient navigation”[Mesh] OR “patient navigator”[Title/Abstract] OR “peer counselor”[Title/Abstract] OR “outreach worker”[Title/Abstract] OR “health promoter”[Title/Abstract] OR “CHW”[Title/Abstract] OR “community-based health worker*”[Title/Abstract] OR “community healthcare workers”[Title/Abstract] OR “community health volunteer”[Title/Abstract] OR “community-based agent”[Title/Abstract] OR “health extension worker”[Title/Abstract] OR “community health agent”[Title/Abstract] OR “health surveillance assistant”[Title/Abstract] OR “ community health promoters” OR “rural health worker”[Title/Abstract] OR “barefoot doctor”[Title/Abstract] OR “backpack health worker”[Title/Abstract] OR “community-based nutrition worker*”[Title/Abstract] OR “accredited social health activist”[Title/Abstract] OR “social health activist”[Title/Abstract] OR “multi-purpose health workers”[Title/Abstract] OR “rural health motivators”[Title/Abstract] OR “village health helper”[Title/Abstract] OR “community health officer”[Title/Abstract] OR “lay counselor”[Title/Abstract] OR “community link workers”[Title/Abstract] | 16,242 |
| Domain 2: Return on Investment | | |
| #2 | “Return on investment”[All Fields] OR “cost benefit analysis”[All Fields] OR “Cost-effectiveness”[All Fields] OR “ROI”[Title/Abstract] OR “Cost*”[Title/Abstract] OR “Economic*”[Title/Abstract] OR “cost analysis”[Title/Abstract] OR “Return*”[Title/Abstract] OR “Rate of return”[Title/Abstract] OR “Payback”[Title/Abstract] OR “Cost reduction”[Title/Abstract] OR “cost saving”[Title/Abstract] OR “saving*”[Title/Abstract] OR “Value for money” | 1,601,712 |
| Domain 3: United states | | |
| #3 | “United States”[MeSH] OR “United States of America”[MeSH] OR “United state*”[Title/Abstract] OR “United states” [Title/Abstract] “United states of America” [Title/Abstract] OR “USA” [Title/Abstract] OR “America” OR “Alabama” OR “Alaska” OR “Arizona” OR “Arkansas” OR “California” OR “Colorado” OR “Connecticut” OR “Delaware” OR “Florida” OR “Georgia” OR “Hawaii” OR “Idaho” OR “Illinois” OR “Indiana” OR “Iowa” OR “Kansas” OR “Kentucky” OR “Louisiana” OR “Maine” OR “Maryland” OR “Massachusetts” OR “Michigan” OR “Minnesota” OR “Mississippi” OR “Missouri” OR “Montana” OR “Nebraska” OR “Nevada” OR “New Hampshire” OR “New Jersey” OR “New Mexico” OR “New York” OR “North Carolina” OR “North Dakota” OR “Ohio” OR “Oklahoma” OR “Oregon” OR “Pennsylvania” OR “Rhode Island” OR “South Carolina” OR “South Dakota” OR “Tennessee” OR “Texas” OR “Utah” OR “Vermont” OR “Virginia” OR “Washington” OR “West Virginia” OR “Wisconsin” OR “Wyoming” | 6,749,983 |
| #4 | #1 AND #2 AND #3 | 1,241 |
| #5 | “Review”[Publication Type] OR “systematic review”[Publication Type] OR “Narrative Review”[Title] OR “Systematic review”[Title] | 3,648,815 |
| #6 | #4 NOT #5 | 1,112 |

## S1B. Embase

| **No.** | **Search strategy** | **No. of Hits** |
| --- | --- | --- |
| Domain 1: Community Health Worker | | |
| #1 | 'health auxiliary'/exp OR 'health auxiliary' | 11208 |
| #2 | 'community health worker'/exp OR 'community health worker' | 11,766 |
| #3 | 'community health worker' OR 'community health worker[ti,ab]' OR 'community health workers' OR 'community health work*' OR 'community health aide*' OR 'village health worker*' OR 'village-based family planning worker[ti,ab]' OR 'lay health*' OR 'lay health worker[ti,ab]' OR 'health coach[ti,ab]' OR 'health aide[ti,ab]' OR 'health educator[ti,ab]' OR 'patient navigation[ti,ab]' OR 'patient navigator[ti,ab]' OR 'peer counselor[ti,ab]' OR 'outreach worker[ti,ab]' OR 'health promoter[ti,ab]' OR 'chw[ti,ab]' OR 'community-based health worker*[ti,ab]' OR 'community healthcare workers[ti,ab]' OR 'community health volunteer[ti,ab]' OR 'community-based agent[ti,ab]' OR 'health extension worker[ti,ab]' OR 'community health agent[ti,ab]' OR 'health surveillance assistant[ti,ab]' OR 'community health promoters' OR 'rural health worker[ti,ab]' OR 'barefoot doctor[ti,ab]' OR 'backpack health worker[ti,ab]' OR 'community-based nutrition worker*[ti,ab]' OR 'accredited social health activist[ti,ab]' OR 'social health activist[ti,ab]' OR 'multi-purpose health workers[ti,ab]' OR 'rural health motivators[ti,ab]' OR 'village health helper[ti,ab]' OR 'community health officer[ti,ab]' OR 'lay counselor[ti,ab]' OR 'community link workers[ti,ab]' | 11,013 |
| #4 | #1 OR #2 OR #3 | 15,266 |
| Domain 2: Return on Investment | | |
| #5 | 'return on investment'/exp OR 'return on investment' | 3,697 |
| #6 | 'cost benefit analysis' OR 'cost-effectiveness' OR 'roi[ti,ab]' OR 'cost*[ti,ab]' OR 'economic*[ti,ab]' OR 'cost analysis[ti,ab]' OR 'return*[ti,ab]' OR 'rate of return[ti,ab]' OR 'payback[ti,ab]' OR 'cost reduction[ti,ab]' OR 'cost saving[ti,ab]' OR 'saving*[ti,ab]' OR 'value for money' | 315,468 |
| #7 | #5 OR #6 | 318,136 |
| Domain 3: United states | | |
| #8 | 'united states':ab,kw,ti,ff | 496,677 |
| #9 | 'united states[ti,ab]' OR 'united states of america[ti,ab]' OR 'united state*[ti,ab]' OR 'usa [ti,ab]' OR 'america[ti,ab]' OR 'alabama' OR 'alaska' OR 'arizona' OR 'arkansas' OR 'california' OR 'colorado' OR 'connecticut' OR 'delaware' OR 'florida' OR 'georgia' OR 'hawaii' OR 'idaho' OR 'illinois' OR 'indiana' OR 'iowa' OR 'kansas' OR 'kentucky' OR 'louisiana' OR 'maine' OR 'maryland' OR 'massachusetts' OR 'michigan' OR 'minnesota' OR 'mississippi' OR 'missouri' OR 'montana' OR 'nebraska' OR 'nevada' OR 'new hampshire' OR 'new jersey' OR 'new mexico' OR 'new york' OR 'north carolina' OR 'north dakota' OR 'ohio' OR 'oklahoma' OR 'oregon' OR 'pennsylvania' OR 'rhode island' OR 'south carolina' OR 'south dakota' OR 'tennessee' OR 'texas' OR 'utah' OR 'vermont' OR 'virginia' OR 'washington' OR 'west virginia' OR 'wisconsin' OR 'wyoming' | 9,087,814 |
| #10 | #8 OR #9 | 9,295,331 |
| #11 | #4 AND #7 AND #10 | 283 |

## S1C: Web of Science

| **No.** | **Search strategy** | **No. of Hits** |
| --- | --- | --- |
| Domain 1: Community Health Worker | | |
| #1 | TS=("community health worker" OR "community health worker*" OR "community health workers" OR "community health work*" OR "community health aide*" OR "village health worker*" OR "village-based family planning worker" OR "lay health*" OR "lay health worker" OR "health coach" OR "health aide" OR "health educator" OR "patient navigation" OR "patient navigator" OR "peer counselor" OR "outreach worker" OR "health promoter" OR "chw" OR "community-based health worker*" OR "community healthcare workers" OR "community health volunteer" OR "community-based agent" OR "health extension worker" OR "community health agent" OR "health surveillance assistant" OR "community health promoters" OR "rural health worker" OR "barefoot doctor" OR "backpack health worker" OR "community-based nutrition worker*" OR "accredited social health activist" OR "social health activist" OR "multi-purpose health workers" OR "rural health motivators" OR "village health helper" OR "community health officer" OR "lay counselor" OR "community link workers") | 14772 |
| Domain 2: Return on Investment | | |
| #2 | TS=("return of investment" OR "cost benefit analysis" OR "cost-effectiveness" OR "ROI" OR "cost*" OR "economic*" OR "cost analysis" OR "return*" OR "rate of return" OR "payback" OR "cost reduction" OR "cost saving" OR "saving*" OR "value for money") | 5,098,202 |
| Domain 3: United states | | |
| #3 | TS=("united states" OR "united states of America" OR "united state*" OR "USA" OR "America" OR "Alabama" OR "Alaska" OR "Arizona" OR "Arkansas" OR "California" OR "Colorado" OR "Connecticut" OR "Delaware" OR "Florida" OR "Georgia" OR "Hawaii" OR "Idaho" OR "Illinois" OR "Indiana" OR "Iowa" OR "Kansas" OR "Kentucky" OR "Louisiana" OR "Maine" OR "Maryland" OR "Massachusetts" OR "Michigan" OR "Minnesota" OR "Mississippi" OR "Missouri" OR "Montana" OR "Nebraska" OR "Nevada" OR "New Hampshire" OR "New Jersey" OR "New Mexico" OR "New York" OR "North Carolina" OR "North Dakota" OR "Ohio" OR "Oklahoma" OR "Oregon" OR "Pennsylvania" OR "Rhode Island" OR "South Carolina" OR "South Dakota" OR "Tennessee" OR "Texas" OR "Utah" OR "Vermont" OR "Virginia" OR "Washington" OR "West Virginia" OR "Wisconsin" OR "Wyoming") | 2,877,786 |
| #4 | #1 AND #2 AND #3 | 539 |

## S1D: EconLit (Via EBSCOhost)

| **No.** | **Search strategy** | **No. of Hits** |
| --- | --- | --- |
| #1 | ("community health worker" OR "community health worker*" OR "community health workers" OR "community health work*" OR "community health aide*" OR "village health worker*" OR "village-based family planning worker" OR "lay health*" OR "lay health worker" OR "health coach" OR "health aide" OR "health educator" OR "patient navigation" OR "patient navigator" OR "peer counselor" OR "outreach worker" OR "health promoter" OR "chw" OR "community-based health worker*" OR "community healthcare workers" OR "community health volunteer" OR "community-based agent" OR "health extension worker" OR "community health agent" OR "health surveillance assistant" OR "community health promoters" OR "rural health worker" OR "barefoot doctor" OR "backpack health worker" OR "community-based nutrition worker*" OR "accredited social health activist" OR "social health activist" OR "multi-purpose health workers" OR "rural health motivators" OR "village health helper" OR "community health officer" OR "lay counselor" OR "community link workers") | 173 |
| #2 | ("return of investment" OR "cost benefit analysis" OR "cost-effectiveness" OR "ROI" OR "cost*" OR "economic*" OR "cost analysis" OR "return*" OR "rate of return" OR "payback" OR "cost reduction" OR "cost saving" OR "saving*" OR "value for money") | 1,54,030 |
| #3 | #1 AND #2 | 168 |

## S1E: CEA Registry by Tufts

| **No.** | **Search strategy** | **No. of Hits** |
| --- | --- | --- |
| #1 | ("community health worker" OR "community health worker*" OR "community health workers" OR "community health work*" OR "community health aide*" OR "village health worker*" OR "village-based family planning worker" OR "lay health*" OR "lay health worker" OR "health coach" OR "health aide" OR "health educator" OR "patient navigation" OR "patient navigator" OR "peer counselor" OR "outreach worker" OR "health promoter" OR "chw" OR "community-based health worker*" OR "community healthcare workers" OR "community health volunteer" OR "community-based agent" OR "health extension worker" OR "community health agent" OR "health surveillance assistant" OR "community health promoters" OR "rural health worker" OR "barefoot doctor" OR "backpack health worker" OR "community-based nutrition worker*" OR "accredited social health activist" OR "social health activist" OR "multi-purpose health workers" OR "rural health motivators" OR "village health helper" OR "community health officer" OR "lay counselor" OR "community link workers") | 42380 |
| #2 | ("return of investment" OR "cost benefit analysis" OR "cost-effectiveness" OR "ROI" OR "cost*" OR "economic*" OR "cost analysis" OR "return*" OR "rate of return" OR "payback" OR "cost reduction" OR "cost saving" OR "saving*" OR "value for money") | 42412 |
| #3 | ("united states" OR "united states of America" OR "united state*" OR "USA" OR "America" OR "Alabama" OR "Alaska" OR "Arizona" OR "Arkansas" OR "California" OR "Colorado" OR "Connecticut" OR "Delaware" OR "Florida" OR "Georgia" OR "Hawaii" OR "Idaho" OR "Illinois" OR "Indiana" OR "Iowa" OR "Kansas" OR "Kentucky" OR "Louisiana" OR "Maine" OR "Maryland" OR "Massachusetts" OR "Michigan" OR "Minnesota" OR "Mississippi" OR "Missouri" OR "Montana" OR "Nebraska" OR "Nevada" OR "New Hampshire" OR "New Jersey" OR "New Mexico" OR "New York" OR "North Carolina" OR "North Dakota" OR "Ohio" OR "Oklahoma" OR "Oregon" OR "Pennsylvania" OR "Rhode Island" OR "South Carolina" OR "South Dakota" OR "Tennessee" OR "Texas" OR "Utah" OR "Vermont" OR "Virginia" OR "Washington" OR "West Virginia" OR "Wisconsin" OR "Wyoming") | 25417 |
| #4 | #1 AND #2 AND #3 | 46 |

# Supplementary file S2: Detailed description of CHW activities organized under the seven major role categories.

| **Categories** | **Activities under each role** |
| --- | --- |
| 1. Bridging/cultural mediation between communities and the health care systems | - Educating community members about health care and social service systems - Educating health and social service systems about community needs and perspectives:   - Changing services offered   - Changing service delivery   - Changing attitudes and behaviors - Information gathering - Interpretation and translation |
| 1. Providing culturally appropriate and accessible health education and information | - Teaching concepts of health promotion and disease prevention - Helping to manage chronic illness - Training other community health advisors |
| 1. Assuring that people get the services they need | - Case finding - Making referrals - Motivating and encouraging people to obtain care - Taking people to services - Providing follow-up |
| 1. Providing informal counseling and social support | - Providing individual support and informal counseling - Leading support groups |
| 1. Advocating for individual and community needs | - Acting as spokespersons for clients - Acting as intermediaries between clients and the health and social service systems - Advocating for the needs and perspectives of communities |
| 1. Providing direct services | - Providing clinical services   - Administering basic first aid   - Administering screening tests (i.e., heights and weights, vision, hearing, and dental screening; blood pressure; temperature; blood glucose) - Meeting basic needs (i.e., assuring that people have the basic determinants of good health, such as food, adequate housing, clothing, and employment) |
| 1. Building individual and community capacity | - Building individual capacity - Building community capacity - Assessing individual and community needs |

Adapted from: Rosenthal EL, et al., J Ambul Care Manage 2011; 34(3): 247-59.

# Supplementary file S3: List of excluded studies (N=69)

| **Reasons for exclusion** | **List of studies** |
| --- | --- |
| Not CHW (n=6) | 1. Bryant-Stephens T, Williams Y, Kanagasundaram J, Apter A, Kenyon CC, Shults J. The West Philadelphia asthma care implementation study (NHLBI# U01HL138687). Contemporary Clinical Trials Communications. 2021 Dec 1;24:100864. 2. Martin MA, Zimmerman LJ, Rosales GF, Lee HH, Songthangtham N, Pugach O, Sandoval AS, Avenetti D, Alvarez G, Gansky SA. Design and sample characteristics of COordinated Oral health Promotion (CO-OP) Chicago: A cluster-randomized controlled trial. Contemporary clinical trials. 2020 May 1;92:105919. 3. Gerber BS, Rapacki L, Castillo A, Tilton J, Touchette DR, Mihailescu D, Berbaum ML, Sharp LK. Design of a trial to evaluate the impact of clinical pharmacists and community health promoters working with African-Americans and Latinos with diabetes. BMC Public Health. 2012 Oct 23;12(1):891. 4. Patel MI, Moore D, Coker TR. End-of-life cancer care redesign: Patient and caregiver experiences in a lay health worker–led intervention. American Journal of Hospice and Palliative Medicine®. 2019 Dec;36(12):1081-8. 5. Pugh LC, Milligan RA, Frick KD, Spatz D, Bronner Y. Breastfeeding duration, costs, and benefits of a support program for low‐income breastfeeding women. Birth. 2002 Jun;29(2):95-100. 6. Knuttgen H, Buffone P, Fowkes E. An innovative approach to reducing home health aide supply costs. Home care manager. 1998;2(4):27-8. |
| Not ROI (n=32) | 1. Freeman AL, Li T, Kaplan SA, Ellen IG, Young A, Rubin D, Gourevitch MN, Doran KM. A pilot community health worker program in subsidized housing: The health+ housing project. Cityscape. 2018 Jan 1;20(2):19-38. 2. Rizzo E, Pascha VM, Gilardino R, Belk K. EPH7 Racial Disparities in Colorectal Cancer Outcomes: How Community Health Workers Can Increase Screening Uptake in the US. Value in Health. 2022 Jul 1;25(7):S435. 3. Shireman TI, Adia AC, Tan Y, Zhu L, Rhee J, Ogunwobi OO, Ma GX. Online versus in-person training of community health workers to enhance hepatitis B virus screening among Korean Americans: Evaluating cost & outcomes. Preventive Medicine Reports. 2020 Sep 1;19:101131. 4. Lapidos A, Lapedis J, Heisler M. Realizing the value of community health workers—new opportunities for sustainable financing. New England Journal of Medicine. 2019 May 23;380(21):1990-2. 5. Panjrath GS, Bostrom L, Al-Saleh Q, Robie S, Baute S, Rhein M, Katz R. COMMUNITY HEALTH WORKERS REDUCE READMISSIONS IN A HIGH RISK HEART FAILURE POPULATION. Journal of the American College of Cardiology. 2016 Apr 5;67(13S):1434-. 6. Rosenthal EL, de Heer H, Rush CH, Holderby LR. Focus on the future: a community health worker research agenda by and for the field. Prog Community Health Partnersh. 2008 Fall;2(3):183-184, 225-35. doi: 10.1353/cpr.0.0025. PMID: 20208200. 7. Andrews JO, Felton G, Wewers ME, Heath J. Use of community health workers in research with ethnic minority women. J Nurs Scholarsh. 2004;36(4):358-65. doi: 10.1111/j.1547-5069.2004.04064.x. PMID: 15636417. 8. Gary TL, Batts-Turner M, Bone LR, Yeh HC, Wang NY, Hill-Briggs F, Levine DM, Powe NR, Hill MN, Saudek C, McGuire M, Brancati FL. A randomized controlled trial of the effects of nurse case manager and community health worker team interventions in urban African-Americans with type 2 diabetes. Control Clin Trials. 2004 Feb;25(1):53-66. doi: 10.1016/j.cct.2003.10.010. PMID: 14980748. 9. Sabo S, Allen CG, Sutkowi K, Wennerstrom A. Community Health Workers in the United States: Challenges in Identifying, Surveying, and Supporting the Workforce. Am J Public Health. 2017 Dec;107(12):1964-1969. doi: 10.2105/AJPH.2017.304096. Epub 2017 Oct 19. PMID: 29048953; PMCID: PMC5678391. 10. Obasanjo I, Griffin M, Scott A, Oberoi S, Westhoff C, Shelton P, Toney S. A Case Study of a Community Health Worker Program Located in Low-Income Housing in Richmond, Virginia. J Community Health. 2022 Apr;47(2):316-323. doi: 10.1007/s10900-021-01057-1. Epub 2022 Jan 10. PMID: 35001203; PMCID: PMC8743086. 11. Anugu M, Braksmajer A, Huang J, Yang J, Ladowski KL, Pati S. Enriched Medical Home Intervention Using Community Health Worker Home Visitation and ED Use. Pediatrics. 2017 May;139(5):e20161849. doi: 10.1542/peds.2016-1849. Epub 2017 Apr 13. PMID: 28557721. 12. Mays, G. P., and H. Felix. "USING COMMUNITY HEALTH WORKERS AND CONSUMER CHOICE TO REBALANCE LONG-TERM CARE AND COSTS IN ARKANSAS." *GERONTOLOGIST*. Vol. 50. JOURNALS DEPT, 2001 EVANS RD, CARY, NC 27513 USA: OXFORD UNIV PRESS INC, 2010. 13. Kwan BM, Rockwood A, Bandle B, Fernald D, Hamer MK, Capp R. Community Health Workers: Addressing Client Objectives Among Frequent Emergency Department Users. J Public Health Manag Pract. 2018 Mar/Apr;24(2):146-154. doi: 10.1097/PHH.0000000000000540. PMID: 28141671; PMCID: PMC5794249. 14. Prentiss, Tyler, et al. "Community health workers as innovators: methods and results from a tele-education pilot for community health workers in Detroit, Michigan." (2017). 15. Freeman AL, Li T, Kaplan SA, Ellen IG, Gourevitch MN, Young A, Doran KM. Community Health Worker Intervention in Subsidized Housing: New York City, 2016-2017. Am J Public Health. 2020 May;110(5):689-692. doi: 10.2105/AJPH.2019.305544. Epub 2020 Mar 19. PMID: 32191526; PMCID: PMC7144437. 16. Carter J, Swack N, Isselbacher E, Donelan K, Thorndike A. Feasibility, Acceptability, and Preliminary Effectiveness of a Combined Digital Platform and Community Health Worker Intervention for Patients With Heart Failure: Protocol for a Randomized Controlled Trial. JMIR Res Protoc. 2024 Feb 6;13:e55687. doi: 10.2196/55687. PMID: 38216543; PMCID: PMC10879973. 17. Ponce-Gonzalez IM, Jimenez N, Rodriguez E, Srivastava A, Parchman ML. Community Health Worker-led Implementation of the Stanford Youth Diabetes Coaching Program in Underserved Latinx Communities. J Prim Care Community Health. 2023 Jan-Dec;14:21501319231158285. doi: 10.1177/21501319231158285. PMID: 36905316; PMCID: PMC10009028. 18. Burns ME, Galbraith AA, Ross-Degnan D, Balaban RB. Feasibility and evaluation of a pilot community health worker intervention to reduce hospital readmissions. Int J Qual Health Care. 2014 Aug;26(4):358-65. doi: 10.1093/intqhc/mzu046. Epub 2014 Apr 16. PMID: 24744082; PMCID: PMC4542649. 19. Islam NS, Wyatt LC, Patel SD, Shapiro E, Tandon SD, Mukherji BR, Tanner M, Rey MJ, Trinh-Shevrin C. Evaluation of a community health worker pilot intervention to improve diabetes management in Bangladeshi immigrants with type 2 diabetes in New York City. Diabetes Educ. 2013 Jul-Aug;39(4):478-93. doi: 10.1177/0145721713491438. Epub 2013 Jun 7. PMID: 23749774; PMCID: PMC3912744. 20. Breysse J, Dixon S, Gregory J, Philby M, Jacobs DE, Krieger J. Effect of weatherization combined with community health worker in-home education on asthma control. Am J Public Health. 2014 Jan;104(1):e57-64. doi: 10.2105/AJPH.2013.301402. Epub 2013 Nov 14. PMID: 24228661; PMCID: PMC3910032. 21. Cardarelli R, Horsley M, Ray L, Maggard N, Schilling J, Weatherford S, Feltner F, Gilliam K. Reducing 30-day readmission rates in a high-risk population using a lay-health worker model in Appalachia Kentucky. Health Educ Res. 2018 Feb 1;33(1):73-80. doi: 10.1093/her/cyx064. PMID: 29474535. 22. Boldt A, Nguyen M, King S, Breitenstein SM. Community Health Workers: Connecting Communities and Supporting School Nurses. NASN Sch Nurse. 2021 Mar;36(2):99-103. doi: 10.1177/1942602X20976545. Epub 2020 Dec 14. PMID: 33307960. 23. Gu KD, Cheng J, Malone A, Faulkner KC, Bejarano O, Gelsomin E, Thorndike AN. Patient and Community Health Worker (CHW) Perspectives on a CHW-delivered Nutrition Intervention for Low-Income Adults with Hypertension: A Qualitative Study. J Prim Care Community Health. 2024 Jan-Dec;15:21501319241285855. doi: 10.1177/21501319241285855. PMID: 39374104; PMCID: PMC11462560. 24. Carter J, Walton A, Donelan K, Thorndike A. Implementing community health worker-patient pairings at the time of hospital discharge: A randomized control trial. Contemp Clin Trials. 2018 Nov;74:32-37. doi: 10.1016/j.cct.2018.09.013. Epub 2018 Oct 4. PMID: 30291997. 25. Gunderson JM, Wieland ML, Quirindongo-Cedeno O, Asiedu GB, Ridgeway JL, OʼBrien MW, Nelson TM, Buzard R, Campbell C, Njeru JW. Community Health Workers as an Extension of Care Coordination in Primary Care: A Community-Based Cosupervisory Model. J Ambul Care Manage. 2018 Oct/Dec;41(4):333-340. doi: 10.1097/JAC.0000000000000255. PMID: 30015685; PMCID: PMC6112848. 26. Keegan CN, Johnston CA, Cardenas VJ Jr, Vaughan EM. Evaluating the Impact of Telehealth-Based, Diabetes Medication Training for Community Health Workers on Glycemic Control. J Pers Med. 2020 Sep 11;10(3):121. doi: 10.3390/jpm10030121. PMID: 32932865; PMCID: PMC7564547. 27. Godecker AL, Harrison PA, Sidebottom AC. Nurse versus community health worker identification of psychosocial risks in pregnancy through a structured interview. J Health Care Poor Underserved. 2013 Nov;24(4):1574-85. doi: 10.1353/hpu.2013.0164. PMID: 24185153. 28. Babamoto KS, Sey KA, Camilleri AJ, Karlan VJ, Catalasan J, Morisky DE. Improving diabetes care and health measures among hispanics using community health workers: results from a randomized controlled trial. Health Educ Behav. 2009 Feb;36(1):113-26. doi: 10.1177/1090198108325911. PMID: 19188371. 29. Tan MM, Villamar DM, Huard C, Nicholson L, Medina HN, Moreno PI. Advance Care Planning With Black Women with Breast Cancer: A Community Health Worker Model. Cancer Control. 2023 Jan-Dec;30:10732748231162479. doi: 10.1177/10732748231162479. PMID: 36916318; PMCID: PMC10020146. 30. Witmer A, Seifer SD, Finocchio L, Leslie J, O'Neil EH. Community health workers: integral members of the health care work force. Am J Public Health. 1995 Aug;85(8 Pt 1):1055-8. doi: 10.2105/ajph.85.8_pt_1.1055. PMID: 7625495; PMCID: PMC1615805. 31. Rodriguez A, Chavez L, Wagner T, Howe C. Effectiveness of Trained Community Lay Workers on Glycemic Control, Knowledge, and Self-Efficacy Among Agricultural Workers with Diabetes in the Texas Panhandle. J Immigr Minor Health. 2024 Oct;26(5):841-849. doi: 10.1007/s10903-024-01603-8. 32. Lockhart E, Turner D, Martinez-Tyson D, Baldwin JA, Marhefka SL. Opportunities for and Perceptions of Integrating Community Health Workers Via the Affordable Care Act: Medicaid Health Homes. J Public Health Manag Pract. 2021 Mar-Apr 01;27(2):193-200. doi: 10.1097/PHH.0000000000001118. |
| Not USA(n=1) | 1. World Health Organization. Community Health Worker Programmes In The WHO African Region: Evidence and Options — Policy Brief. Available at <https://www.afro.who.int/sites/default/files/2017-07/Community%20Health%20Worker%20Policy%20Brief%20-%20English_0.pdf> |
| Review (n=1) | 1. Norris SL, Chowdhury FM, Van Le K, et al. Effectiveness of community health workers in the care of persons with diabetes. *Diabet Med*. 2006;23(5):544-556. doi:10.1111/j.1464-5491.2006.01845.x |
| Insufficient information on ROI (N=29) | 1. Smith L, Atherly A, Campbell J, Flattery N, Coronel S, Krantz M. Cost-effectiveness of a statewide public health intervention to reduce cardiovascular disease risk. BMC Public Health. 2019 Sep 6;19(1):1234. doi: 10.1186/s12889-019-7573-8. PMID: 31492118; PMCID: PMC6728976. 2. Johnston, K., M. Meier, and M. Federico. "A Community Health Worker Led Home Visit Program Integrated into Asthma Specialty Care Decreases Health Care Utilization and Shows a Sustained Impact on Asthma Control." *B102. DISPARITIES IN ASTHMA MANAGEMENT*. American Thoracic Society, 2019. A4061-A4061. 3. Piatt, Gretchen, et al. "Cost-Effectiveness of Diabetes Self-Management Education and Support in the Community—Projections from a Randomized Controlled Trial." *Diabetes* 67.Supplement_1 (2018): 703-P. 4. Thompson MP, Podila PSB, Clay C, Sharp J, Bailey-DeLeeuw S, Berkley AJ, Baker BG, Waters TM. Community navigators reduce hospital utilization in super-utilizers. Am J Manag Care. 2018 Feb;24(2):70-76. PMID: 29461853. 5. The Cost-Effectiveness of Community Health Workers: A Chronic Kidney Disease Markov Model 6. Schechter CB, Walker EA, Ortega FM, Chamany S, Silver LD. Costs and effects of a telephonic diabetes self-management support intervention using health educators. J Diabetes Complications. 2016 Mar;30(2):300-5. doi: 10.1016/j.jdiacomp.2015.11.017. Epub 2015 Nov 24. PMID: 26750743; PMCID: PMC4761277. 7. Allen JK, Dennison Himmelfarb CR, Szanton SL, Frick KD. Cost-effectiveness of nurse practitioner/community health worker care to reduce cardiovascular health disparities. J Cardiovasc Nurs. 2014 Jul;29(4):308-14. doi: 10.1097/JCN.0b013e3182945243. PMID: 23635809; PMCID: PMC3766479. 8. Rush CH. Return on investment from employment of community health workers. J Ambul Care Manage. 2012 Apr-Jun;35(2):133-7. doi: 10.1097/JAC.0b013e31822c8c26. PMID: 22415287. 9. Fedder DO, Chang RJ, Curry S, Nichols G. The effectiveness of a community health worker outreach program on healthcare utilization of west Baltimore City Medicaid patients with diabetes, with or without hypertension. Ethn Dis. 2003 Winter;13(1):22-7. PMID: 12723008. 10. Knowles M, Crowley AP, Vasan A, Kangovi S. Community Health Worker Integration with and Effectiveness in Health Care and Public Health in the United States. Annu Rev Public Health. 2023 Apr 3;44:363-381. doi: 10.1146/annurev-publhealth-071521-031648. PMID: 37010928. 11. Embick ER, Maeng DD, Juskiewicz I, Cerulli C, Crean HF, Wittink M, Poleshuck E. Demonstrated health care cost savings for women: findings from a community health worker intervention designed to address depression and unmet social needs. Arch Womens Ment Health. 2021 Feb;24(1):85-92. doi: 10.1007/s00737-020-01045-9. Epub 2020 Jun 16. PMID: 32548774; PMCID: PMC9305631. 12. Roth AM, Holmes AM, Stump TE, Aalsma MC, Ackermann RT, Carney TS, Katz BP, Kesterson J, Erdman SM, Balt CA, Inui TS. Can lay health workers promote better medical self-management by persons living with HIV? An evaluation of the Positive Choices program. Patient Educ Couns. 2012 Oct;89(1):184-90. doi: 10.1016/j.pec.2012.06.010. Epub 2012 Jul 5. PMID: 22770948. 13. Mirambeau AM, Wang G, Ruggles L, Dunet DO. A cost analysis of a community health worker program in rural Vermont. J Community Health. 2013 Dec;38(6):1050-7. doi: 10.1007/s10900-013-9713-x. PMID: 23794072; PMCID: PMC4602368. 14. Heisler M, Lapidos A, Kieffer E, Henderson J, Guzman R, Cunmulaj J, Wolfe J, Meyer T, Ayanian JZ. Impact on Health Care Utilization and Costs of a Medicaid Community Health Worker Program in Detroit, 2018-2020: A Randomized Program Evaluation. Am J Public Health. 2022 May;112(5):766-775. doi: 10.2105/AJPH.2021.306700. Epub 2022 Mar 24. PMID: 35324259; PMCID: PMC9010898. 15. Carter J, Hassan S, Walton A, Yu L, Donelan K, Thorndike AN. Effect of Community Health Workers on 30-Day Hospital Readmissions in an Accountable Care Organization Population: A Randomized Clinical Trial. JAMA Netw Open. 2021 May 3;4(5):e2110936. doi: 10.1001/jamanetworkopen.2021.10936. PMID: 34014324; PMCID: PMC8138690. 16. Zare H, Delgado P, Spencer M, Thorpe RJ Jr, Thomas L, Gaskin DJ, Werrell LK, Carter EL. Using Community Health Workers to Address Barriers to Participation and Retention in Diabetes Prevention Program: A Concept Paper. J Prim Care Community Health. 2022 Jan-Dec;13:21501319221134563. doi: 10.1177/21501319221134563. PMID: 36331112; PMCID: PMC9638527. 17. Rice K, Sharma K, Li C, Butterly L, Gersten J, DeGroff A. Cost-effectiveness of a patient navigation intervention to increase colonoscopy screening among low-income adults in New Hampshire. Cancer. 2019 Feb 15;125(4):601-609. doi: 10.1002/cncr.31864. Epub 2018 Dec 12. PMID: 30548480; PMCID: PMC6399743. 18. Frick KD, Pugh LC, Milligan RA. Costs related to promoting breastfeeding among urban low-income women. J Obstet Gynecol Neonatal Nurs. 2012 Jan-Feb;41(1):144-150. doi: 10.1111/j.1552-6909.2011.01316.x. Epub 2011 Dec 12. PMID: 22151148. 19. Moffett ML, Kaufman A, Bazemore A. Community Health Workers Bring Cost Savings to Patient-Centered Medical Homes. J Community Health. 2018 Feb;43(1):1-3. doi: 10.1007/s10900-017-0403-y. PMID: 28695425; PMCID: PMC5767191. 20. Shih YC, Chien CR, Moguel R, Hernandez M, Hajek RA, Jones LA. Cost-Effectiveness Analysis of a Capitated Patient Navigation Program for Medicare Beneficiaries with Lung Cancer. Health Serv Res. 2016 Apr;51(2):746-67. doi: 10.1111/1475-6773.12333. Epub 2015 Jun 26. PMID: 26119569; PMCID: PMC4799903. 21. Molina Y, Pichardo CM, Patrick DL, Ramsey SD, Bishop S, Beresford SAA, Coronado GD. Estimating the costs and cost-effectiveness of promoting mammography screening among US-based Latinas. J Health Dispar Res Pract. 2018;12(6):10. PMID: 34414017; PMCID: PMC8373201. 22. Patel MI, Kapphahn K, Wood E, Coker T, Salava D, Riley A, Krajcinovic I. Effect of a Community Health Worker-Led Intervention Among Low-Income and Minoritized Patients With Cancer: A Randomized Clinical Trial. J Clin Oncol. 2024 Feb 10;42(5):518-528. doi: 10.1200/JCO.23.00309. Epub 2023 Aug 25. Erratum in: J Clin Oncol. 2024 Apr 20;42(12):1457. doi: 10.1200/JCO.24.00286. PMID: 37625110. 23. Crespo R, Christiansen M, Tieman K, Wittberg R. An Emerging Model for Community Health Worker-Based Chronic Care Management for Patients With High Health Care Costs in Rural Appalachia. Prev Chronic Dis. 2020 Feb 13;17:E13. doi: 10.5888/pcd17.190316. PMID: 32053481; PMCID: PMC7021460. 24. Ballard M, Johnson A, Mwanza I, Ngwira H, Schechter J, Odera M, Mbewe DN, Moenga R, Muyingo P, Jalloh R, Wabwire J, Gichaga A, Choudhury N, Maru D, Keronyai P, Westgate C, Sapkota S, Olsen HE, Muther K, Rapp S, Raghavan M, Lipman-White K, French M, Napier H, Nepomnyashchiy L. Community Health Workers in Pandemics: Evidence and Investment Implications. Glob Health Sci Pract. 2022 Apr 29;10(2):e2100648. doi: 10.9745/GHSP-D-21-00648. PMID: 35487542; PMCID: PMC9053152. 25. Patel MI, Sundaram V, Desai M, Periyakoil VS, Kahn JS, Bhattacharya J, Asch SM, Milstein A, Bundorf MK. Effect of a Lay Health Worker Intervention on Goals-of-Care Documentation and on Health Care Use, Costs, and Satisfaction Among Patients With Cancer: A Randomized Clinical Trial. JAMA Oncol. 2018 Oct 1;4(10):1359-1366. doi: 10.1001/jamaoncol.2018.2446. PMID: 30054634; PMCID: PMC6233780. 26. Cross-Barnet, Caitlin, et al. "Higher quality at lower cost: Community health worker interventions in the health care innovation awards." *Journal of Health Disparities Research and Practice* 11.2 (2018): 10. 27. Coutinho MT, Subzwari SS, McQuaid EL, Koinis-Mitchell D. Community Health Workers' Role in Supporting Pediatric Asthma Management: A Review. Clin Pract Pediatr Psychol. 2020 Jun;8(2):195-210. doi: 10.1037/cpp0000319. PMID: 35498877; PMCID: PMC9053383. 28. Perry, Henry, and Rose Zulliger. "How effective are community health workers." An overview of current evidence with recommendations for strengthening community health worker programs to accelerate progress in achieving the health-related Millennium Development Goals. Baltimore: Johns Hopkins Bloomberg School of Public Health 84 (2012). 29. Ye W, Kuo S, Kieffer EC, Piatt G, Sinco B, Palmisano G, Spencer MS, Herman WH. Cost-Effectiveness of a Diabetes Self-Management Education and Support Intervention Led by Community Health Workers and Peer Leaders: Projections From the Racial and Ethnic Approaches to Community Health Detroit Trial. Diabetes Care. 2021 May;44(5):1108-1115. doi: 10.2337/dc20-0307. |

# Supplementary file S4: The quality assessment of included studies

| **Author, Year** | **Q1** | **Q2** | **Q3** | **Q4** | **Q5** | **Q6** | **Q7** | **Q8** | **Q9** | **Q10** | **Q11** | **Q12** | **Q13** | **Q14** | **Q15** | **Q16** | **Q17** | **Total**  **(Out of 17)** |
| --- | --- | --- | --- | --- | --- | --- | --- | --- | --- | --- | --- | --- | --- | --- | --- | --- | --- | --- |
| Kangovi S 2020 | 1 | 1 | 1 | 1 | 1 | 1 | 1 | 1 | 0 | 0 | 1 | 1 | 1 | 1 | 1 | 1 | 0 | 14 |
| London K 2017 | 1 | 1 | 1 | 1 | 1 | 1 | 1 | 1 | 0 | 0 | 1 | 1 | 1 | 1 | 1 | 1 | 0 | 14 |
| Willink A 2020 | 1 | 1 | 1 | 1 | 1 | 1 | 1 | 1 | 0 | 0 | 1 | 1 | 1 | 1 | 1 | 1 | 0 | 14 |
| Marshall ET 2020 | 1 | 1 | 1 | 1 | 1 | 1 | 1 | 1 | 0 | 0 | 1 | 1 | 1 | 0 | 1 | 1 | 0 | 13 |
| Ryabov I 2014 | 1 | 1 | 1 | 0 | 1 | 1 | 1 | 1 | 0 | 1 | 1 | 1 | 1 | 1 | 1 | 1 | 0 | 14 |
| van der Goes DN 2019 | 1 | 1 | 1 | 0 | 1 | 1 | 1 | 1 | 1 | 1 | 1 | 1 | 1 | 1 | 1 | 1 | 0 | 15 |
| Whitley EM 2006 | 1 | 1 | 1 | 0 | 1 | 0 | 0 | 0 | 1 | 0 | 0 | 1 | 0 | 0 | 1 | 1 | 0 | 8 |
| Wilson FA 2015 | 1 | 1 | 1 | 0 | 1 | 1 | 1 | 1 | 0 | 1 | 1 | 1 | 1 | 1 | 1 | 1 | 1 | 15 |
| Patel MI 2020 | 1 | 1 | 1 | 0 | 1 | 1 | 1 | 1 | 0 | 0 | 0 | 0 | 0 | 0 | 1 | 1 | 0 | 9 |
| Cramer ME 2018 | 1 | 1 | 1 | 1 | 1 | 1 | 1 | 1 | 1 | 0 | 1 | 1 | 1 | 1 | 1 | 1 | 0 | 15 |
| Cardarelli R 2018 | 1 | 1 | 1 | 1 | 1 | 1 | 1 | 1 | 0 | 0 | 1 | 1 | 1 | 0 | 1 | 1 | 0 | 13 |
| Naufal G 2022 | 1 | 1 | 1 | 0 | 1 | 1 | 1 | 1 | 0 | 0 | 1 | 1 | 1 | 1 | 1 | 1 | 0 | 13 |
| Morgan AU 2016 | 1 | 0 | 1 | 1 | 1 | 1 | 1 | 1 | 0 | 0 | 1 | 1 | 1 | 0 | 1 | 1 | 0 | 12 |
| Bhaumik U 2020 | 1 | 1 | 1 | 1 | 1 | 1 | 1 | 1 | 0 | 0 | 1 | 1 | 1 | 0 | 1 | 1 | 0 | 13 |
| Vohra AS  2020 | 1 | 1 | 1 | 1 | 1 | 1 | 1 | 1 | 1 | 0 | 0 | 1 | 1 | 0 | 1 | 1 | 0 | 13 |
| Turcotte DA 2014 | 1 | 1 | 1 | 0 | 0 | 1 | 1 | 1 | 0 | 0 | 0 | 1 | 1 | 0 | 1 | 1 | 0 | 10 |
| Brown HS 2012 | 1 | 1 | 1 | 1 | 1 | 1 | 1 | 1 | 0 | 1 | 0 | 1 | 1 | 1 | 1 | 1 | 0 | 14 |
| Ohuabunwa U 2021 | 1 | 1 | 1 | 0 | 1 | 1 | 1 | 1 | 1 | 0 | 0 | 1 | 1 | 0 | 1 | 1 | 0 | 12 |
| Campbell JD 2015 | 1 | 1 | 1 | 1 | 1 | 1 | 1 | 1 | 0 | 0 | 1 | 1 | 1 | 1 | 1 | 1 | 0 | 14 |
| Huang SJ 2019 | 1 | 1 | 1 | 1 | 1 | 1 | 1 | 1 | 0 | 0 | 0 | 1 | 1 | 1 | 1 | 1 | 0 | 13 |
| Margellos-Anast H 2012 | 1 | 1 | 1 | 1 | 1 | 1 | 1 | 1 | 0 | 0 | 1 | 1 | 1 | 0 | 1 | 1 | 0 | 13 |
| Enard KR 2013 | 1 | 1 | 1 | 0 | 1 | 1 | 1 | 1 | 1 | 0 | 0 | 1 | 1 | 0 | 1 | 1 | 0 | 12 |
| Li Y 2017 | 1 | 1 | 1 | 1 | 1 | 1 | 1 | 1 | 0 | 1 | 0 | 1 | 1 | 1 | 1 | 1 | 0 | 14 |
| Fiori KP 2024 | 0 | 0 | 1 | 0 | 0 | 1 | 1 | 1 | 0 | 0 | 0 | 1 | 1 | 0 | 1 | 1 | 0 | 8 |
| Mechanic OJ  2022 | 1 | 1 | 1 | 0 | 1 | 1 | 1 | 1 | 1 | 0 | 1 | 1 | 1 | 1 | 1 | 1 | 0 | 14 |
| Lawlor MS 2013 | 1 | 1 | 1 | 1 | 0 | 0 | 1 | 1 | 0 | 0 | 0 | 1 | 1 | 0 | 1 | 1 | 0 | 10 |
| Herman PM  2022 | 1 | 1 | 1 | 1 | 1 | 1 | 1 | 1 | 0 | 0 | 0 | 1 | 1 | 0 | 1 | 1 | 0 | 12 |
| Garson A Jr 2012 | 1 | 1 | 1 | 1 | 1 | 1 | 1 | 1 | 0 | 0 | 0 | 1 | 1 | 0 | 1 | 1 | 0 | 12 |
| Galbraith AA  2017 | 1 | 1 | 1 | 0 | 1 | 1 | 1 | 1 | 1 | 0 | 0 | 1 | 1 | 0 | 1 | 1 | 0 | 12 |
| Corliss J 2015 | 0 | 1 | 1 | 0 | 1 | 1 | 1 | 1 | 1 | 0 | 0 | 1 | 1 | 0 | 1 | 1 | 0 | 11 |
| Lightner JS  2025 | 1 | 1 | 1 | 0 | 1 | 1 | 1 | 1 | 0 | 0 | 0 | 1 | 1 | 0 | 1 | 1 | 0 | 11 |
| Fawcett KJ Jr 2018 | 1 | 1 | 1 | 0 | 1 | 1 | 1 | 1 | 0 | 0 | 1 | 1 | 1 | 0 | 1 | 1 | 0 | 12 |
| Christiansen E 2017 | 1 | 1 | 1 | 1 | 1 | 1 | 1 | 1 | 1 | 0 | 1 | 1 | 1 | 0 | 1 | 1 | 0 | 14 |
| McCarthy D 2020 | 1 | 1 | 1 | 1 | 1 | 1 | 1 | 1 | 1 | 0 | 1 | 1 | 1 | 1 | 1 | 1 | 0 | 15 |
| Gillam P 2020 | 1 | 1 | 1 | 1 | 1 | 1 | 1 | 1 | 1 | 0 | 1 | 1 | 1 | 1 | 1 | 1 | 0 | 15 |
| Average | | | | | | | | | | | | | | | | | | 12.66 / 17 |

**The leading questions in the quality assessment (Q1-Q17)**

Adapted from: <https://www.nice.org.uk/process/pmg4/chapter/appendix-i-quality-appraisal-checklist-economic-evaluations#cba-checklist-for-compilers-of-nice-public-health-reviews>

1. Is there a well-defined question?
2. Is there a comprehensive description of alternatives?
3. Was one of the alternatives designated as the comparator against which the intervention was evaluated?
4. Is the perspective stated?
   - Is WTP the public-sector WTP or the aggregated individual WTP? Has the WTP been recalibrated when the basis for its calculation has not coincided with the perspective being used?
5. Are all important and relevant costs and outcomes for each alternative identified?

- Check to see if the study is of money-costs and 'benefits' which are savings of future money-costs.

1. Has effectiveness been established?
2. Are costs and outcomes measured accurately?
3. Are costs and outcomes valued credibly?
4. Have all important and relevant costs and outcomes for each alternative been quantified in money terms?
   - If not, state which items were not quantified, and the likely extent of their importance in terms of influencing the benefit: cost ratio.
5. Are costs and outcomes adjusted for differential timing?
6. Has at least one of Net Present Value, B:C ratio and payback period been estimated?
7. Were any assumptions of materiality made?
8. Were all assumptions reasonable in the circumstances in which they were made, and were they justified?
9. Were sensitivity analyses conducted to investigate uncertainty in estimates of cost or benefits?
10. How far do study results include all issues of concern to users?
11. Are the results generalisable to the setting of interest in the review?
    - Country differences.
    - Question of interest differs from the CBA question being reviewed.
12. Have equity considerations been addressed in any way?

# Supplementary file S5: The intervention cost, total net cost-saving and the ROI

| **Author, year** | **Reported by studies** | | | **CPI Conversion ratio (as per 2024 USD)** | **Recalculated based on the average annual CPI (2024 USD)** | | |
| --- | --- | --- | --- | --- | --- | --- | --- |
|  | **Total cost of Intervention (per year)** | **Total net cost saving (per year)** | **ROI** |  | **Total cost of Intervention (per year)** | **Total net cost saving (per year)** | **ROI** |
| Kangovi S 2020 | 567,950.82 | 1,401,307.99 | 2.47 | 1.21 | 688405.29 | 1698505.92 | 2.47 |
| London K 2017 (Asthma) | 76333 | 142333 | 1.86 | 1.28 | 81218.31 | 151442.31 | 1.86 |
| London K 2017 (T2DM) | 145,000 | 162400 | 1.12 | 1.28 | 154280.00 | 172793.60 | 1.12 |
| London K 2017 (Complex disease) | 314667 | 755200 | 2.40 | 1.28 | 334805.69 | 803532.80 | 2.40 |
| London K 2017 (CVD complications) | 64667 | 129333 | 2.00 | 1.28 | 68805.69 | 137610.31 | 2.00 |
| Willink A 2020 | 451440 | 505613 | 1.12 | 1.21 | 460468.80 | 515725.26 | 1.12 |
| Marshall ET 2020 | 38,731 | 51,900 | 1.34 | 1.21 | 39505.62 | 52938.00 | 1.34 |
| Ryabov I 2014 | 7681 | 13,810 | 1.80 | 1.33 | 10177.33 | 18298.25 | 1.80 |
| van der Goes DN 2019 | 955523 | 1133290 | 1.19 | 1.23 | 986099.74 | 1169555.28 | 1.19 |
| Whitley EM 2006 | 74753 | 170437 | 2.28 | 1.58 | 118184.49 | 269460.90 | 2.28 |
| Wilson FA 2015 | 185,357 | 424,760 | 2.29 | 1.32 | 245227.31 | 561957.48 | 2.29 |
| Patel MI 2020 | 765,000 | 3,080,400 | 4.03 | 1.21 | 927180.00 | 3733444.80 | 4.03 |
| Cramer ME 2018 | 123,615 | 260,268 | 2.11 | 1.25 | 154395.14 | 325074.73 | 2.11 |
| Cardarelli R 2018 | 39,936 | -26,846 | -0.67 | 1.25 | 49880.06 | -33530.65 | -0.67 |
| Naufal G 2022 | 28,885 | 457,204 | 15.83 | 1.07 | 30964.72 | 490122.69 | 15.83 |
| Morgan AU 2016 | 125000 | 225,000 | 1.80 | 1.31 | 163375.00 | 294075.00 | 1.80 |
| Bhaumik U 2020 | 15750 | 21578 | 1.37 | 1.21 | 19089.00 | 26152.54 | 1.37 |
| Vohra AS 2020 | 128,115 | 128,115 | 1.00 | 1.21 | 155275.38 | 155275.38 | 1.00 |
| Turcotte DA 2014 | 32,640 | 38522 | 1.18 | 1.33 | 43248.00 | 51041.65 | 1.18 |
| Brown HS 3rd, 2012 | 274709 | 1112572 | 4.05 | 1.38 | 380746.67 | 1542024.79 | 4.05 |
| Ohuabunwa U 2021 | 350,000 | 7,512,883 | 21.47 | 1.16 | 405300.00 | 8699918.51 | 21.47 |
| Campbell JD 2015 | 108,878 | 206,514 | 1.90 | 1.32 | 144045.59 | 273218.02 | 1.90 |
| Huang SJ 2019 | 35227 | 106737 | 3.03 | 1.23 | 43223.53 | 130966.30 | 3.03 |
| Margellos-Anast H 2012 | 22,953 | 128,080 | 5.58 | 1.38 | 31812.86 | 177518.88 | 5.58 |
| Enard KR 2013 | 45,880 | 504,680 | 11.00 | 1.35 | 61800.36 | 679803.96 | 11.00 |
| Li Y 2017 | 466605 | 4120122 | 8.83 | 1.28 | 597254.40 | 5273756.16 | 8.83 |
| Fiori KP 2024 | 515,992 | 1,274,501 | 2.47 | 1.00 | 515992.00 | 1274501.00 | 2.47 |
| Mechanic OJ 2022 | 71512 | 117060 | 1.64 | 1.07 | 76660.86 | 125488.32 | 1.64 |
| Lawlor MS 2013 | 63750 | 141525 | 2.22 | 1.35 | 85871.25 | 190634.18 | 2.22 |
| Herman PM 2022 | 19060 | 36215 | 1.90 | 1.07 | 20432.32 | 38822.48 | 1.90 |
| Garson A Jr 2012 | 40,503 | 91,008 | 2.25 | 1.38 | 56137.16 | 126137.09 | 2.25 |
| Galbraith AA 2017 | 432768 | 1284000 | 2.97 | 1.28 | 553943.04 | 1643520.00 | 2.97 |
| Corliss J 2015 | 7500000 | 118050000 | 15.74 | 1.32 | 10200000.00 | 160548000.00 | 15.74 |
| Lightner JS 2025 | 454942.6667 | 6125772.333 | 13.46 | 1.00 | 454942.67 | 6125772.33 | 13.46 |
| Fawcett KJ Jr 2018 | 552,636 | 757,111 | 1.37 | 1.25 | 690242.36 | 945631.64 | 1.37 |
| Christiansen E 2017 | 278,331 | 503,384 | 1.81 | 1.28 | 356263.68 | 644331.52 | 1.81 |
| McCarthy D 2020 | 1,440,720 | 2,478,038 | 1.72 | 1.21 | 1746152.64 | 3003382.06 | 1.72 |
| Gillam P 2020 (Health Access) | 79500 | 772725 | 9.72 | 1.21 | 96354.00 | 936542.70 | 9.72 |
| Gillam P 2020 (Birth Matters) | 156,668 | 332754 | 2.12 | 1.21 | 189881.62 | 403297.85 | 2.12 |
| Gillam P 2020 (Prisma Health Upstate) | 551,983 | 3,400,000 | 6.16 | 1.21 | 669003.40 | 4120800.00 | 6.16 |
| Gillam P 2020 (Tandem Health) | 140,044 | 92865 | 0.66 | 1.21 | 169733.33 | 112552.38 | 0.66 |
| **Average (Standard deviation)** | | | | | 543,326.32 (1,565,032.34) | 5,063,660.89 (24,654,276.81) | 4.11 (4.81) |
| **Median (Interquartile range)** | | | | | 155,275.38 (61800.36 to 460468.80) | 403,297.85 (137610.31 to 1274501.00) | 2.12 (1.64 to 4.03) |

CPI: Consumer price index; ROI: return on investment; USD: United States Dollars

# Supplementary file S6: Factors affecting the ROI

| **Factors** | | **Median ROI (IQR)** | **95% CI** | **P value** |
| --- | --- | --- | --- | --- |
| Perspectives | |  |  | 0.598 |
|  | Societal (n=4) | 3.54 (2.68) | - |  |
|  | Payer (n=23) | 2.12 (4.32) |  |  |
|  | Provider (n=14) | 1.95 (0.70) | - |  |
| Regions | |  |  | 0.260 |
|  | South (n=16) | 3.14 (8.00) | - |  |
|  | Northeast (n=12) | 1.83 (1.05) | - |  |
|  | West (n=7) | 1.90 (0.80) | - |  |
|  | Midwest (n=6) | 1.91 (3.25) | - |  |
| Target Population | |  |  | 0.378 |
|  | Diabetes (n=8) | 1.93 (1.53) | - |  |
|  | Asthma (n=7) | 1.37 (4.88) | - |  |
|  | High risk of readmission (n=7) | 2.00 (9.00) | - |  |
|  | Others (n=19) | 2.21 (4.00) | - |  |
| Programme Duration | |  |  | 0.940 |
|  | < 1 year (n=14) | 2.06 (1.10) | - |  |
|  | ≥ 1 year (n=27) | 2.00 (2.86) | - |  |
| Sensitivity analysis | |  |  | 0.491 |
|  | Yes (n=17) | 2.06 (4.38) | - |  |
|  | No (n=24) | 2.00 (1.33) | - |  |

CI: Confidence interval; IQR: Interquartile range; ROI: Return on investment

# Supplementary file S7: Association between CHW roles and ROI

| **CHW Role** | **Number of studies** | **Mean ROI (SD)** | **Median ROI (Range)** | **P-Value** |
| --- | --- | --- | --- | --- |
| **Cultural mediation** | No (n=19) | 3.90 (4.59) | 1.80 (14.83) | 0.472 |
|  | Yes (n=22) | 4.29 (5.21) | 2.23 (22.14) |  |
| **Accessible education and information** | No (n=15) | 3.37 (3.73) | 2.12 (14.13) | 0.587 |
|  | Yes (n=26) | 4.54 (5.45) | 2.16 (20.35) |  |
| **Patient services** | No (n=16) | 4.33 (4.84) | 2.23 (14.71) | 0.556 |
|  | Yes (n=25) | 3.97 (4.99) | 2.00 (22.14) |  |
| **Social support and counselling** | No (n=23) | 5.01 (5.89) | 2.28 (22.14) | 0.220 |
|  | Yes (n=18) | 2.97 (2.94) | 1.88 (10.34) |  |
| **Community advocacy** | No (n=35) | 3.85 (4.86) | 2.11 (22.14) | 0.476 |
|  | Yes (n=6) | 5.66 (5.10) | 4.14 (12.80) |  |
| **Providing direct services** | No (n=18) | 3.00 (3.88) | 1.83 (14.74) | **0.040*** |
|  | Yes (n=23) | 4.98 (5.46) | 2.47 (22.14) |  |
| **Capacity building** | No (n=27) | 4.71 (5.69) | 2.22 (22.14) | 0.610 |
|  | Yes (n=14) | 2.96 (2.50) | 2.06 (9.06) |  |
| **CHW that provide ≥4 roles** | No (n=25) | 3.31 (4.11) | 1.90 (16.50) | 0.209 |
|  | Yes (n=16) | 5.37 (5.80) | 2.43 (20.81) |  |
| **CHW that provide combined direct service and education** | No (n=29) | 3.38 (3.97) | 1.90 (0.99) | 0.058 |
|  | Yes (n=12) | 5.89 (6.65) | 3.0 (5.25) |  |

CHW: Community health worker; ROI: Return on Investment; SD: Standard deviation

*Indicate significance
